# Isolation and growth-promoting mechanisms of phosphate-solubilizing bacteria from Qinghai-Tibet Plateau in *Lespedeza bicolor* Turcz

**DOI:** 10.3389/fmicb.2025.1669774

**Published:** 2025-09-17

**Authors:** Xinru Zhang, Wenjia Liu, Jiayi Liu, Xiaoli Mei, Jinghua Zhang, Dong Wang, Xiaoxia Zhang, Jiayao Zhuang

**Affiliations:** ^1^Collaborative Innovation Center of Sustainable Forestry in Southern China of Jiangsu Province, Nanjing Forestry University, Nanjing, China; ^2^The Third Construction Co., Ltd. of China Construction First Group, Beijing, China; ^3^China Construction First Group Co., Ltd., Beijing, China

**Keywords:** *Bacillus* sp., environmental factor, *Lespedeza bicolor* Turcz, phosphate solubilization, Soil bacterial community

## Abstract

**Introduction:**

The recovery of slopes in high-altitude areas is often challenging due to insufficient nutrients in the soil, with phosphorus deficiency being a key limiting factor for plant growth. This study aimed to screen highly efficient phosphate-solubilizing bacterial (PSB) strains from undisturbed regions of the Qinghai–Tibet Plateau and investigate their growth-promoting effects on *Lespedeza bicolor* Turcz, and explore the optimal configuration and mechanism of bacterial strain–plant combinations.

**Methods:**

Three strains, *Bacillus atrophaeus* (Q4), *B. megaterium* (Q7), and *B. megaterium* (YG1), were obtained through screening experiments.

**Results:**

The results of potted plant experiments showed that the inoculation of the three strains increased the biomass of the seedlings to varying degrees (29.9% - 133.5%) and improved the soil nutrient content and enzyme activity. Among these, Q4 and *L. bicolor* Turcz were a relatively ideal combination, and the Q4 treatment had a better growth-promoting effect (133.5% total biomass increase) compared with the Q7 and YG1 treatments. Compared to the control (CK), inoculation with strain Q4 significantly reduced soil microbial community diversity (*p* < 0.05) and shifted community composition toward dominance by specific taxa. In addition, environmental factors were positively correlated with the abundance of Q4 bacterial strains, indicating that the inoculation of bacterial agents helped improve the release of soil nutrients. The relative abundance of metabolic genes was significantly higher under the Q4 treatment compared with the CK treatment, with metabolism-related products constituting the largest proportion. The abundance of secondary functional genes, such as those related to the metabolism of cofactors and vitamins, carbohydrate metabolism, and amino acid metabolism, increased under the Q4 treatment compared with the CK treatment.

**Conclusion:**

The results suggested that phosphate-solubilizing bacteria could promote the growth of leguminous plants. The study provides a novel approach by leveraging the indirect effects of microbes, that is, increasing soil nutrient content and enzyme activity, to improve the soil environment, which may provide new ideas and methods for ecological restoration in China and similar high-altitude areas in the world.

## 1 Introduction

Phosphorus (P), accounting for approximately 0.2% of plant dry weight, is vital for plant growth and development. The total soil P content is typically high (ranging from 400–1,200 mg kg^−1^); however, less than 1% is bioavailable to plants (Zhu et al., 2018). This limitation is attributed to the predominant existence of organic phosphorus (Po) forms, accounting for 20–80% of the soil P pool (Dalal, 1997). Inorganic phosphorus (Pi), the primary form of phosphorus utilized by plants, presents a global challenge due to its low bioavailability, which results from limited mobility and strong adsorption in soils (Yuefen and Guanghui, 2022). Given its vital role in plant’s metabolic processes (Li et al., 2018), P scarcity consequently severely restricts plant growth (Raymond et al., 2020). P deficiency has become a key limiting factor in vegetation restoration in ecologically fragile regions. A large number of high and steep slopes in northern China generally have characteristics such as thin soil layers, poor nutrients, and poor water retention capacity (Paz-Ares et al., 2022). Traditional engineering measures often lead to vegetation degradation due to insufficient effective P content in soil, aggravating the risk of soil erosion (Li et al., 2020).

Plant growth–promoting rhizobacteria (PGPR) offer an eco-efficient solution to enhance land productivity amid climate change and land degradation (Castagno et al., 2021; Kalayu, 2019; Manuel et al., 2023; Tian et al., 2021). PGPR strains improve nutrient uptake, confer abiotic stress tolerance, and suppress the activity of pathogens, thereby promoting plant growth (Chauhan et al., 2021). Among these, phosphate-solubilizing bacteria (PSB) solubilize or mineralize immobilized soil P (Barra et al., 2018). PSB-mediated P solubilization primarily relies on proton and organic acid secretion, which acidifies the rhizosphere, besides the enzymatic mineralization of organic P via phytases and phosphatases (Alori et al., 2017). Well-characterized PSB genera include *Pseudomonas*, *Bacillus*, *Burkholderia*, and *Rhizobium*, which are known to enhance soil P mobilization (Raymond et al., 2020; Kalayu, 2019; Alori et al., 2017).

Unlike earlier studies focusing on temperate or agricultural soils (Bakhshandeh et al., 2017; Shah et al., 2022), this work investigates PSB from the high-altitude alpine ecosystems of the Qinghai–Tibet Plateau. The Qinghai–Tibet Plateau, an extreme habitat, harbors unique microbial communities adapted to low temperatures, drought, and intense ultraviolet (UV) radiation (Du et al., 2025; Zhang et al., 2024). PSB native to this region may exhibit superior P-solubilizing mechanisms and environmental adaptability (Xie et al., 2024; Zhou et al., 2025), thereby offering novel bioaugmentation strategies for vegetation restoration in alpine barren soils. However, investigations on plateau PSB resources remain limited, and their growth-promoting effects on pioneer plants are poorly understood.

*Lespedeza bicolor* Turcz, a leguminous perennial shrub distributed in mid- to low-altitude mountains of the Qinghai-Tibet Plateau, serves as a key pioneer species for soil conservation. Its stress tolerance (cold, drought, and low-nutrient adaptation) and extensive root-nodule symbioses (Wan et al., 2023) enable ecological resilience. Regarding soil reinforcement, the roots at higher elevations possess greater biomass and tensile strength, making them more resistant to soil erosion under extreme environmental conditions (Qi et al., 2024). However, its growth and ecological functions are often constrained by P deficiency on steep slopes, limiting biomass accumulation and slope stabilization.

In summary, the objectives of this study were to (i) isolate PSB strains from the plateau soils; (ii) evaluate the plant growth–promoting effects of selected PSB strains and identify optimal microbial–plant combinations; and (iii) explore PSB-induced changes in microbial community abundance and structure in the rhizosphere of *L. bicolor* Turcz, while conducting the correlation analysis of soil nutrients and other environmental factors, to elucidate the mechanisms underlying the promoting effect of microbial inoculants on plant growth. This study provides critical insights into utilizing indigenous PSB for ecological restoration in extreme environments, offering a microbial-based solution to enhance soil fertility and improve plant stress resilience under nutrient-limited conditions.

## 2 Materials and methods

### 2.1 Experimental materials

**Figure 1:**
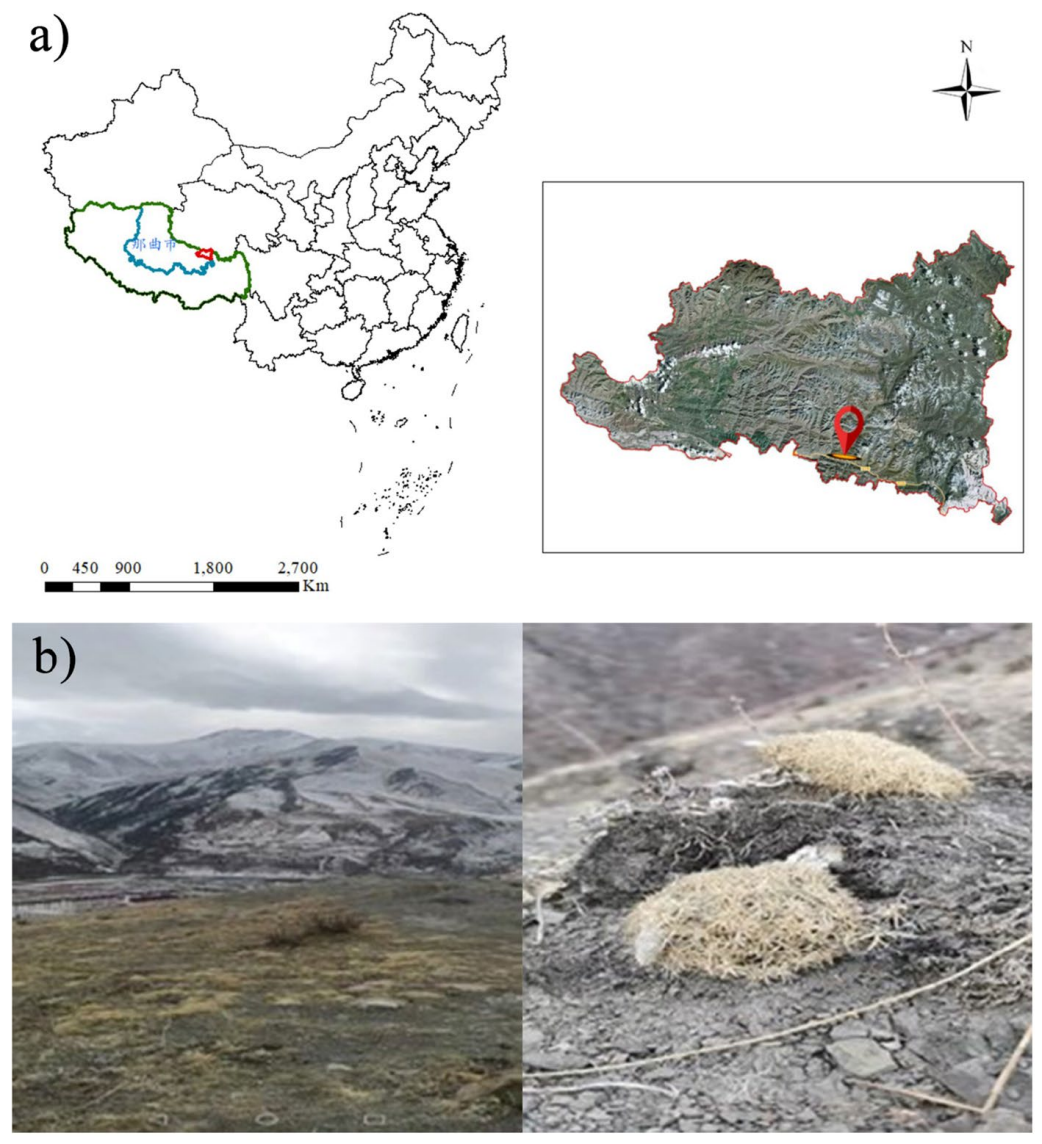
Remote sensing image of the research area **(a)** and the soil source of screened strain **(b)**.

The soil samples for screening microbes were collected from the unmanned interference zone (94°3′9″E, 31°55′56”N) in Baqing County, Nagqu City, Tibet Autonomous Region, China (Figure 1).

The experimental soil was collected from the topsoil (0–15 cm depth) of a farmland at the Baguadao experimental field (118°50′E, 32°11′N) in Qixia District, Nanjing, China. The basic soil contents were as follows: available nitrogen, 26.77 mg kg^−1^; available phosphorus (AP), 28.18 mg kg^−1^; available potassium (AK), 183.79 mg kg^−1^; pH, 7.66; and organic carbon, 15.60 g kg^−1^. The soil was sieved through a 5 × 5 mm mesh to remove debris, including rocks and plant tissues, followed by autoclave sterilization to eliminate plant seeds, soil fauna, and microorganisms.

The preparation of media needed [e.g., Luria broth (LB), *Monkina* organic phosphorus, inorganic phosphate (Pi), Ashby’s nitrogen-free agar, and O-CAS] for microbial screening and functional tests are described in Supplementary materials.

### 2.2 Strain screening and functional analysis

Six soil samples collected from the Qinghai-Tibet Plateau were processed separately. For each sample, 5 g soil was aseptically transferred into a 250 mL conical flask containing 45 mL of sterile distilled water and glass beads. The suspension was vigorously shaken for 20 min at 180 rpm to achieve homogenization. Subsequently, 1 mL of the soil suspension was transferred into a test tube containing 9 mL of sterile physiological saline (0.9% NaCl solution) and mixed thoroughly to obtain a 10^−1^ dilution. This serial dilution process was repeated sequentially to prepare gradient dilutions of 10^−2^ ~ 10^−6^. Aliquots (100 μL) from the 10^−4^, 10^−5^, and 10^−6^ dilutions were spread onto solid LB agar plates supplemented with 2% NaCl (pH 8.0) using sterile glass spreaders. The agar plates were incubated at 28 °C for 6 d. Single colonies were picked and purified by repeated streaking.

*Monkina* organic phosphorus and Pi culture media were used to screen strains with the phosphate-solubilizing effect from the isolated strains. Three replicates were prepared for each strain and then incubated in a constant-temperature incubator at 28 °C for 7 d. The appearance of a transparent circle on the medium indicated that the strains had the phosphate-solubilizing effect. The diameter of the single colony (*d*) and of the transparent circle (*D*) was measured. The value of *D*/*d* was used to evaluate the strength of PSB.

Based on the *D*/*d* values, three strains with a more pronounced phosphate-solubilizing effect were selected for evaluating the phosphate-solubilizing effect of bacteria at different temperatures. The strains exhibiting transparent rings were activated and inoculated into 100 mL of LB liquid medium. The cultures were incubated in a constant-temperature shaker at 30 °C and 200 rpm for 24 h to prepare the seed culture, ensuring that the optical density (OD_600_) of the seed culture was between 0.8 and 1.2 with UV–Vis spectrophotometer (T6, Purkinje General Co., China). Subsequently, 3 mL of the seed culture from each strain was transferred into 250 mL conical flasks containing 100 mL of both *Monkina* organic phosphorus and Pi liquid media. A noninoculated control (CK) was also included. Each strain was tested in triplicate. The cultures were incubated at 0 °C, 4 °C, 16 °C, 22 °C, 30 °C, 35 °C, and 40 °C, with shaking at 200 rpm, for 7 d. After incubation, the cultures were centrifuged at 4 °C for 10 min at 6000 rpm. The supernatants were then analyzed for effective P content using the molybdenum blue colorimetric method, 2 mL of centrifuged supernatant was transferred to a 50 mL volumetric flask. Approximately 20 mL of deionized water was added, followed by one drop of 2,4-dinitrophenol indicator. The solution pH was adjusted to a faint yellow endpoint using 2 mol/L NaOH and 2 mol/L HCl. Then, 5 mL of freshly prepared Molybdenum antimony anti-coloring agent was added using a micropipette. The solution was brought to 50 mL final volume with deionized water, mixed thoroughly, and incubated at room temperature for 30 min. Absorbance was measured at 700 nm using a UV–Vis spectrophotometer. The effective P concentration was determined from a standard phosphorus calibration curve.

Strain derivatives (1-mm-diameter disks) were inoculated on O-CAS medium (5 days) or Ashby’s nitrogen-free agar (6 d) and incubated at 35 ± 0.2 °C (Ustiatik et al., 2021). Positive nitrogen fixation was confirmed by growth on Ashby’s medium (Supplementary Figure S1), while siderophore secretion was indicated by color changes on O-CAS medium (Supplementary Figure S2).

Based on the results of phosphate-solubilizing experiments, nitrogen-fixing experiments, and siderophore secretion experiments, strains Q4, Q7, and YG1 were selected for potting experiments (Supplementary Figure S3) and stored in the Soil and Water Conservation Laboratory of Nanjing Forestry University.

### 2.3 Pot experiment

Three microbial inoculants were fermented in 400 mL of sterile LB liquid medium for 5 h (Ibiang et al., 2020). In the process of fermentation, the value of OD_600_ of the suspension was measured. And make sure the value of is in the range of 0.8 ~ 1.2 by diluting or continuing the fermentation. The bioinoculant was sealed and stored in a refrigerator at 4 °C for later use. At the time of inoculation, the stored bioinoculant was diluted 100 times, and 100 mL of the diluted bioinoculant was applied to the rhizosphere soil for the pot experiment of *L. bicolor* Turcz.

The study was conducted in a greenhouse facility at Nanjing Forestry University, with controlled environmental conditions maintaining an average temperature of 32 °C and relative humidity of 72%. The test soil, collected from agricultural fields at 0–20 cm depth, had the following physicochemical properties: available nitrogen (26.77 mg kg^−1^), available phosphorus (28.18 mg kg^−1^), available potassium (183.79 mg kg^−1^), pH 7.66, and organic carbon content (15.60 g kg^−1^). Prior to experimentation, the soil was air-dried, sieved, and manually cleared of debris. A growth substrate was prepared by mixing soil: vermiculite: perlite at a 3: 1:1 (v/v/v) ratio, followed by sterilization in an autoclave at 120 °C for 15 min. The sterilized substrate was then transferred to cultivation pots (50 cm × 20 cm), with each pot containing 8 L of the prepared growth medium.

*L. bicolor* Turcz seeds were pretreated prior to the pot experiment. First, the seeds were soaked in distilled water for 12 h. The water was then filtered, and the seeds were subsequently soaked in a 0.5% sodium hypochlorite solution for 1 min to disinfect them. Later, the disinfected seeds were washed with pure water and kept in the seedling cup to germinate for 2 weeks. Seedlings of uniform height (6–8 cm) were transplanted into pots at a density of four plants per pot, with 10 cm spacing between plants. Four treatments with 3 replicates were designed, resulting in a total of 12 pots. The main treatments were as follows: (1) sterile water (CK); (2) inoculation with *B. atrophaeus* (Q4); (3) inoculation with *B. megaterium* (Q7); and (4) inoculation with *B. toyonensis* (YG1). During the whole experimental period, potted plants were randomly placed in the greenhouse and rearranged every other month. Identical agronomic management measures were then implemented across all treatments for the study.

The pot experiment was terminated after 3 months, and plant and rhizosphere soil samples were collected for index measurements. For plants, use vernier calipers and tape to measure the ground diameter and height of the seedlings; use a root scanner to measure the root system; and the plants were oven-dried at 60 °C to constant weight (48 ~ 72 h). For potted soil, use the mettler toledo pH meter to determine its pH value (water-soil ratio is 5:1); use the molybdenum-antimony anti-colorimetric method to determine soil AP (Murphy and Riley, 1962); use the Olsen-P method to determine soil available P (Olsen, 1954); use alkali-hydrolyzable nitrogen (HN) diffusion method to determine soil hydrolyzed nitrogen (Walkley and Black, 1934).

In addition, a variety of soil enzyme activities were analyzed. Including soil catalase (S-CAT), soil alkaline phosphatase (S-ALP), soil invertase (S-SC), soil urease (S-UE), and soil dehydrogenase (S-DHA), were analyzed. Each assay included three independent technical replicates per sample. The S-CAT activity was determined by measuring the decomposition of H₂O₂ at 240 nm (Johnson and Temple, 1964). The S-ALP activity was quantified using *p*-nitrophenyl phosphate as substrate by a previously described method (Tabatabai, 1969). S-SC activity was assessed through 3,5-dinitrosalicylic acid colorimetry by measuring the reducing sugar content (Frankeberger and Johanson, 1983). S-UE activity was evaluated by ammonium release using urea as the substrate (Schinner and Mersi, 1990). S-DHA activity was determined by measuring the reduction of triphenyltetrazolium chloride to triphenylformazan at 485 nm (Maachowska-Jutsz and Matyja, 2019).

### 2.4 Extraction and PCR amplification of total soil microbial DNA

Portions of soil samples collected from potted plants were stored at −20 °C. After combining samples from the same treatment into a composite sample, 0.25 g was used for DNA extraction. The entire genomic DNA was isolated using the E.Z.N.A.® Soil DNA Kit (Omega Bio-Tek, GA, USA), following the manufacturer’s guidelines. The concentration of extracted DNA was detected by Nanodrop RND-2000 (NanoDrop Technologies, Wilmington, DE, United States). Post-extraction, the integrity of the DNA was assessed through 1% agarose gel electrophoresis. Then, the universal primers 338F (5’-ACTCCTACGGGAGCAGCAG-3′) and 806R (5’-GGACTACHGGGTWTCTAAT-3′) (Ke et al., 2021) were used to perform PCR amplification and MiSeq sequencing on the V3-V4 region of the bacterial 16SrRNA gene. The PCR reaction consisted of 12.5 μL 2 × Premix Taq™ (TaKaRa. Bio Inc. Shiga, Japan), 1 μL of each primer (10 μm), 2 μL of DNA extract (5–20 ng), and 9 μL of dd H_2_O to a final volume of 25 μL. The PCR amplification conditions as follows: denaturated at 95 °C for 3 min, followed by 35 cycles of denaturation at 95 °C for 30 s, annealing at 55 °C for 30 s, annealing at 55 °C for 30 s, and a final extension step at 72 °C for 10 min. The PCR products of the same samples were mixed and detected by using 2% agarose gel electrophoresis and purified by using an AxyPrepDNA gel recovery kit (Axygen Biosciences, U.S.). Based on the initial electrophoretic assessment, the PCR products’ concentration was measured with a QuantiFluor-ST blue fluorescent quantification system from Promega. The DNA samples were carefully measured and combined in accordance with the specific sequencing requirements for each sample. Sequencing was carried out using Mothur (V.1.36.1). During data processing, columns were cleaned to eliminate chimeric sequences and to refine the final dataset. The steps involved are as follows: initially, FASTP (V.0.19.6) was employed to perform a thorough quality check on the raw sequences, filtering out any low-quality reads. Next, the sequences were assembled into longer reads using FLASH (V.1.2.11) (Edgar et al., 2011; Fukami et al., 2018). Following this, UPARSE (V.11) facilitated the clustering of sequences into operational taxonomic units (OTUs) after quality filtering. Chimeras were identified and removed based on a 97% similarity threshold to ensure the purity of the resulting sequences, which were then categorized into OTUs (Edgar et al., 2011). The high-throughput sequencing of the purified amplicons was performed by Guangzhou Jidi’ao Technology Service Co., Ltd. (Guangzhou, China) using an Illumina MiSeq sequencer (Illumina, CA, USA).

### 2.5 Bioinformatics and statistical analyses

The raw sequence data and quality control files were derived from FASTA format files. According to Schloss, data processing and analysis were carried out using the Mothur software. Microbial diversity in the potted soil, along with the dominance of key species, was assessed through alpha diversity metrics and relative abundance measures such as Chao1, Simpson, and Shannon indices, based on OTU counts. To visualize species composition, the standard ggplot2 package in R was employed, while the vegan package facilitated environmental correlation analyses to explore the links between environmental factors, sample types, and plant types.

The nucleotide sequences of *B. atrophaeus* (Q4), *B. megaterium* (Q7) and *B. toyonensis* (YG1) have been uploaded to the NCBI database (registration numbers: PP733356, PP733357, and PP733360, respectively).

## 3 Results

### 3.1 Phosphate-solubilizing capacity of PSB

**Figure 2:**
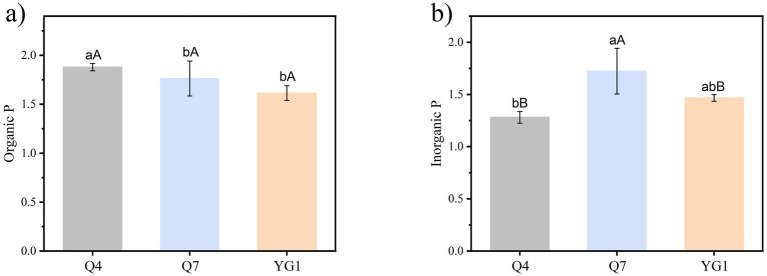
Phosphate-solubilizing index of PSB strains **(a)** Organic P index; **(b)** Inorganic P index. Different lowercase letters indicate significant differences among treatments (*p* < 0.05), while uppercase letters denote significant differences in the ability of the same strain to solubilize Po versus Pi (*p* < 0.05).

The Q4, Q7, and YG1 strains produced obvious transparent phosphate-solubilizing circles on both Po and Pi media (Figure 2). Of these, the Q4 strain had the strongest solubility of Po with a phosphate-solubilizing index of 1.88. Q7 had the strongest solubility of Pi, with a phosphate-solubilizing index of 1.72. The results showed that the three strains could solubilize both Po and Pi, with Q4 and YG1 significantly stronger solubilization ability for Po than for Pi (*p* < 0.05).

**Figure 3:**
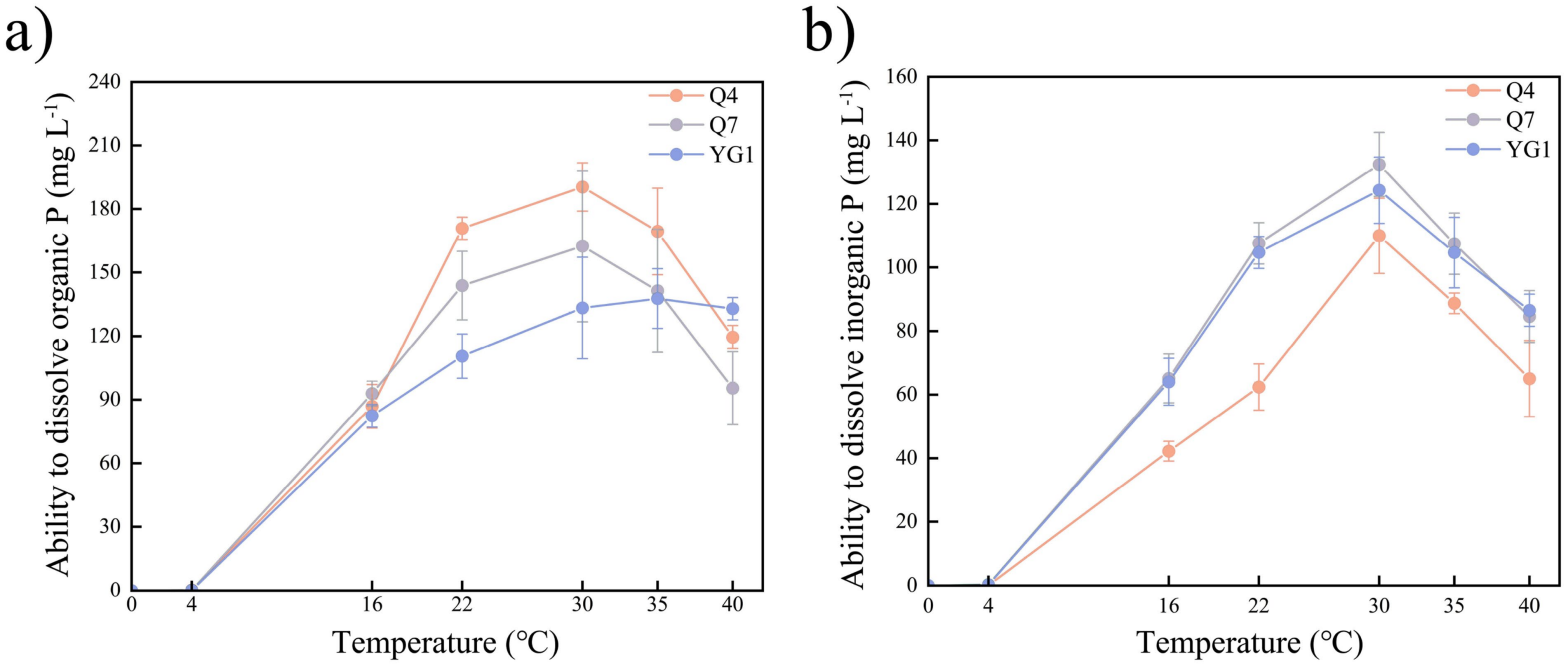
Phosphate-solubilizing index of PSB strains at different temperatures **(a)** Organic P index; **(b)** Inorganic P index.

The phosphate-solubilizing capacity of tested strains was temperature-dependent (Figure 3). While growth was inhibited below 4 °C, all strains grew and solubilized both Po and Pi between 16–40 °C. Strain Q4 showed the highest Po solubilization, peaking at 30 °C, followed by Q7 with similar temperature dependence. The three strains exhibited organic phosphorus solubilization capacities ranging from 110.56–170.80 mg mL^−1^ at 22 °C, Q4 demonstrated a phosphorus solubilization efficiency of 89.72% (relative to 30 °C), which was significantly higher than other strains (*p* < 0.05). For Pi, all strains displayed maximum solubilization at 30 °C, with decreasing efficiency at higher temperatures.

### 3.2 Plant growth

**Figure 4:**
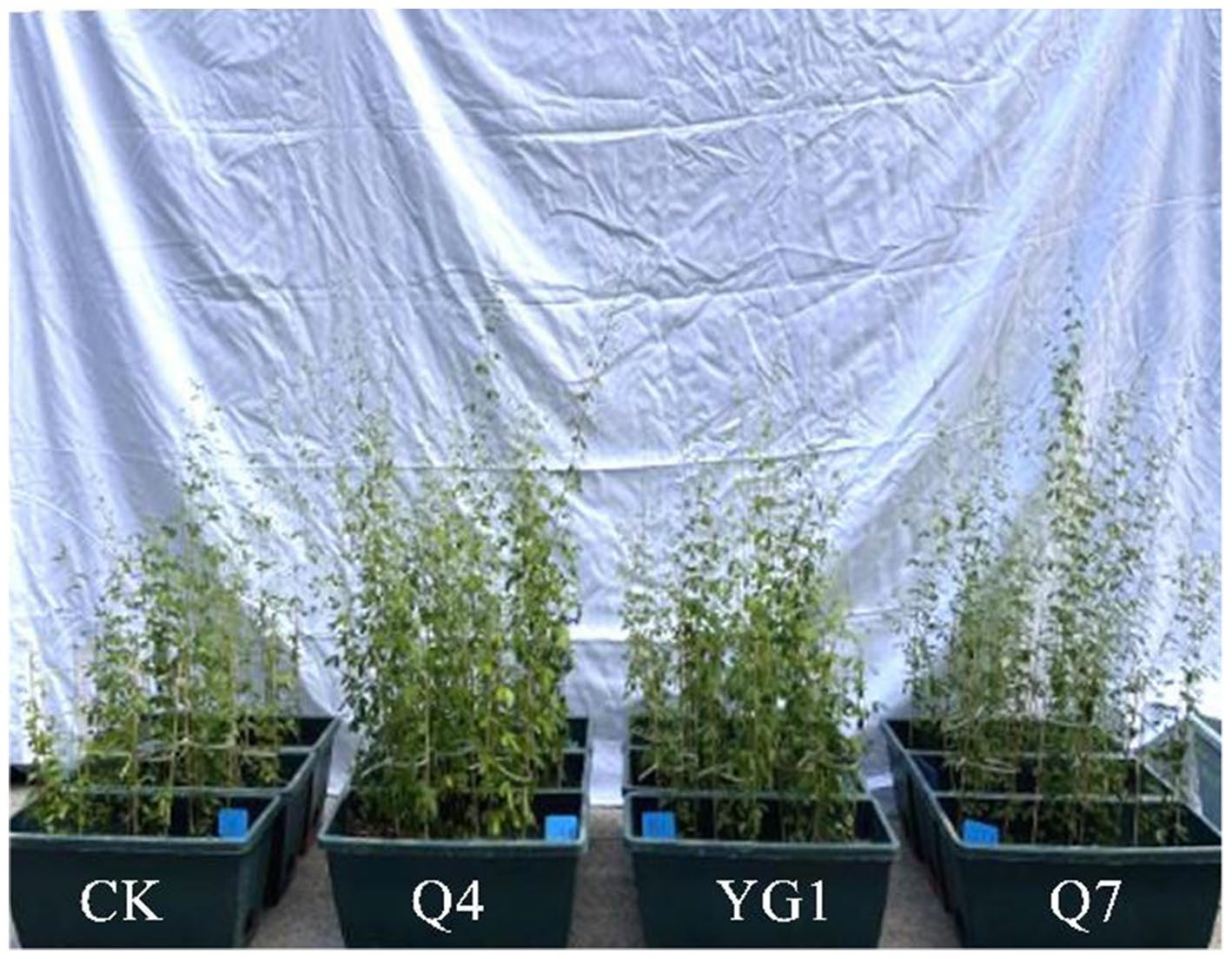
Effects of different treatments on *Lespedeza bicolor* Turcz growth.

**Figure 5:**
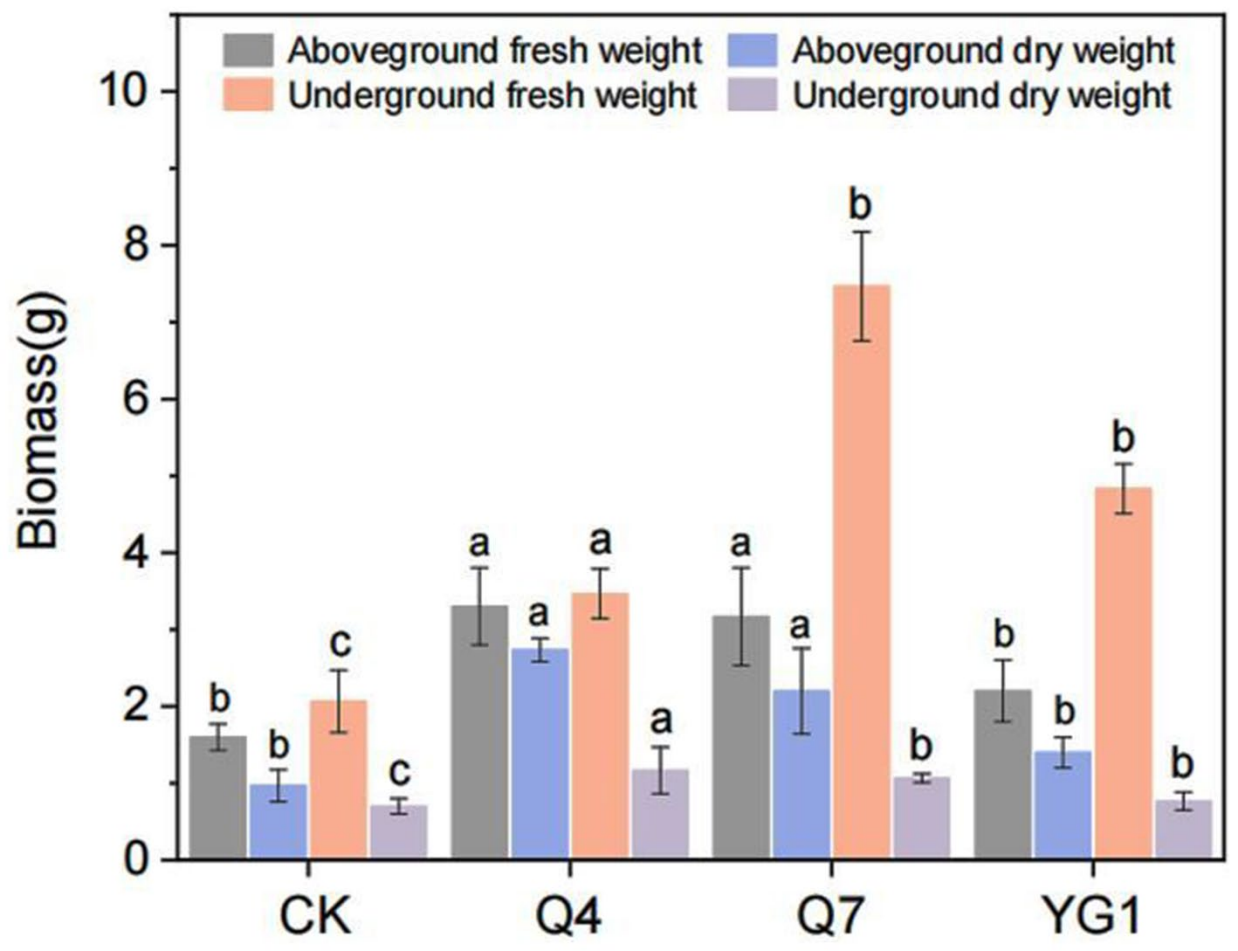
Effects of different treatments on the biomass of *Lespedeza bicolor* Turcz. Letters indicate significant differences (*p* < 0.05) among different treatments.

All inoculated *L. bicolor* Turcz seedlings showed significantly increased biomass compared to CK (Figure 4). Q4 treatment produced the most pronounced enhancement (133.5% total biomass increase), followed by Q7 (95.8%) and YG1 (29.9%) (Figure 5). Aboveground fresh weight increased by 106.3% (Q4), 98.1% (Q7), and 37.5% (YG1), with corresponding dry weight increases of 181.4, 126.8, and 44.3%. Underground portions showed fresh weight gains of 260.9% (Q4), 147.8% (Q7), and 133.3% (YG1), with dry weight increases of 67.1, 52.9, and 10.0%.

All indices related to the root system of *L. bicolor* Turcz showed significant improvements under different microbial treatments (*p* < 0.05) (Table 1). Among these, the Q4 treatment exhibited the most pronounced growth-promoting effects. The root length was 403.99 cm in the Q4 group, which was a significant increase by 206.37% (*p* < 0.05). The root surface area was 45.64 cm^2^, with an increase of 223.28%, and the root volume was 0.41 cm^3^, with an increase of 244.42%. The inoculation effect of Q7 was superior to that of YG1, whereas YG1 outperformed the CK treatment.

**Table 1:** Effects of different microbial agents on the plant roots.

Treatment	Root length (cm)	Root area (cm^2^)	Average diameter (mm)	Root volume (cm^3^)	Tips (pcs)	Forks (pcs)
CK	131.86 ± 22.68b	14.12 ± 2.99b	0.34 ± 0.02a	0.12 ± 0.03c	639 ± 243b	680 ± 138b
Q4	403.99 ± 188.70a	45.64 ± 16.65a	0.37 ± 0.05a	0.41 ± 0.11a	2094 ± 782a	3,815 ± 1834a
Q7	260.65 ± 101.48ab	30.08 ± 9.97ab	0.37 ± 0.04a	0.28 ± 0.08ab	1,446 ± 573ab	1,387 ± 1123b
YG1	221.39 ± 55.07ab	22.84 ± 3.49b	0.34 ± 0.04a	0.19 ± 0.03bc	1,211 ± 359ab	1,283 ± 411b

Univariate analysis of variance, *n* = 3. Different small letters represent significant differences in data between treatment groups (*p* < 0.05). The same below.

Inoculation with PSB significantly enhanced photosynthetic parameters in *L. bicolor* Turcz seedlings (*p* < 0.05, Table 2). Compared to CK, Q4 treatment increased net photosynthetic rate (Pn), stomatal conductance (Gs), and intercellular CO_2_ concentration (Ci) by 94.1, 27.6, and 41.4% respectively, while Q7 and YG1 showed greater improvements (103.3%/52.0%/70.0 and 39.1%/63.1%/77.9%, respectively). Transpiration rate (Tr) and water use efficiency (WUE) were also significantly improved, with Q4 demonstrating the most pronounced effects, followed by Q7 and YG1.

**Table 2:** Effects of different microbial agents on the photosynthetic parameters of seedlings.

Treatment	Pn [μmolCO_2_/(m^2^·s)]	Gs [mmol/(m^2^·s)]	Ci [μmol/(m^2^·s)]	Tr [mmol H_2_O/(m^2^·s)]	WUE [μmolCO_2_/mmol H_2_O]
CK	7.54 ± 1.95c	0.05 ± 0.01c	104 ± 1.00c	1.31 ± 0.30b	12.09 ± 0.06d
Q4	14.63 ± 0.15a	0.10 ± 0.01a	145 ± 10.79b	2.70 ± 0.24a	16.58 ± 0.08a
Q7	9.62 ± 0.28bc	0.08 ± 0.01b	170 ± 13.61a	2.59 ± 0.19a	13.52 ± 0.07b
YG1	10.66 ± 1.01b	0.09 ± 0.02ab	185 ± 16.70a	2.30 ± 0.48a	12.60 ± 0.07c

Different small letters represent significant differences in data between treatment groups (*p* < 0.05).

**Figure 6:**
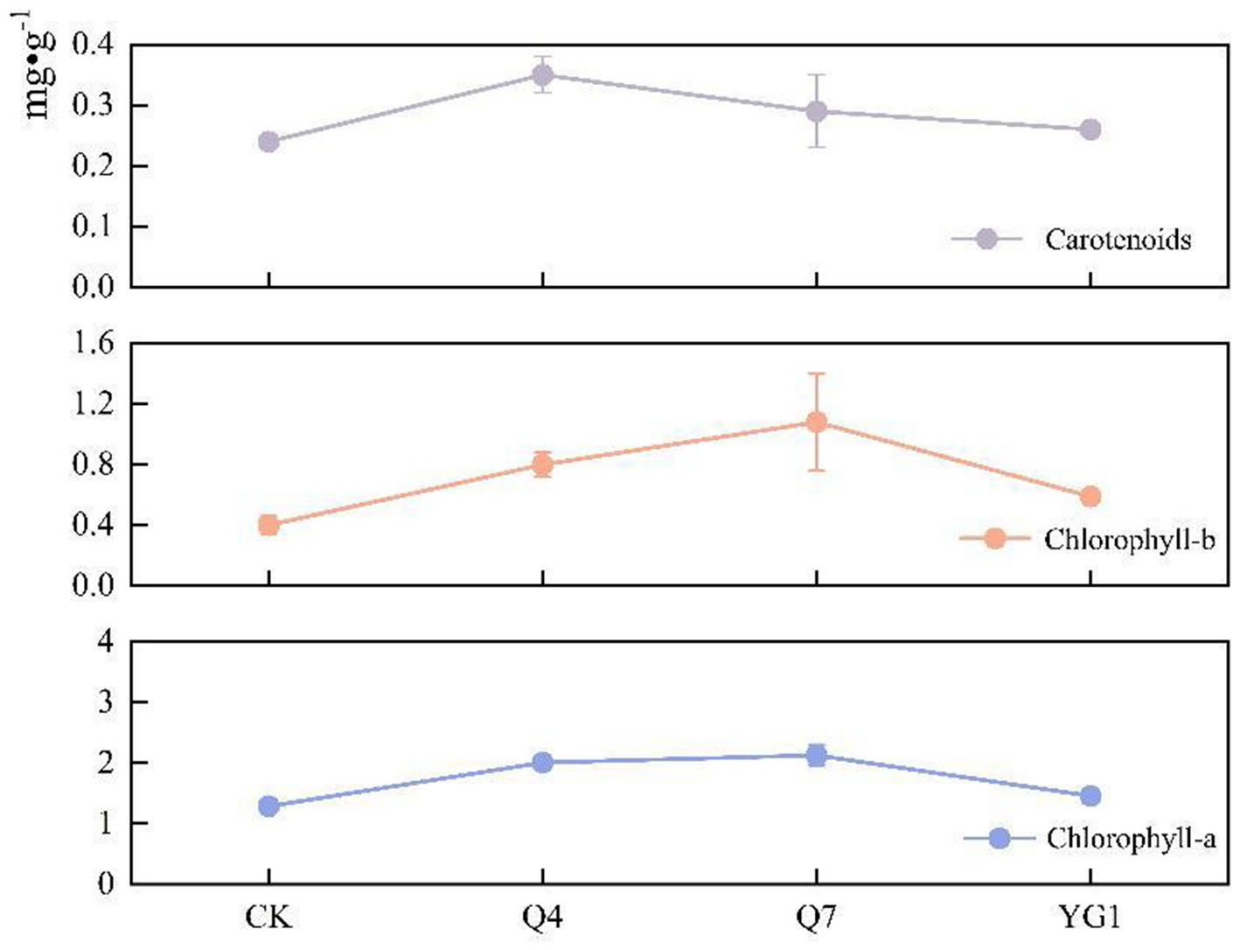
Effects of different treatments on photosynthetic pigments.

Inoculation significantly increased photosynthetic pigment content in *L. bicolor* Turcz seedlings (Figure 6). Compared to CK, chlorophyll a increased by 100.7% (Q4), 113.0% (Q7), and 45.7% (YG1); chlorophyll b by 100.0, 169.2, and 48.3%; and carotenoids by 45.8, 20.8, and 9.7%, respectively. Q7 treatment yielded the highest chlorophyll content, while Q4 showed the greatest carotenoid enhancement.

### 3.3 Soil nutrients, enzyme activities, and P fraction

**Figure 7:**
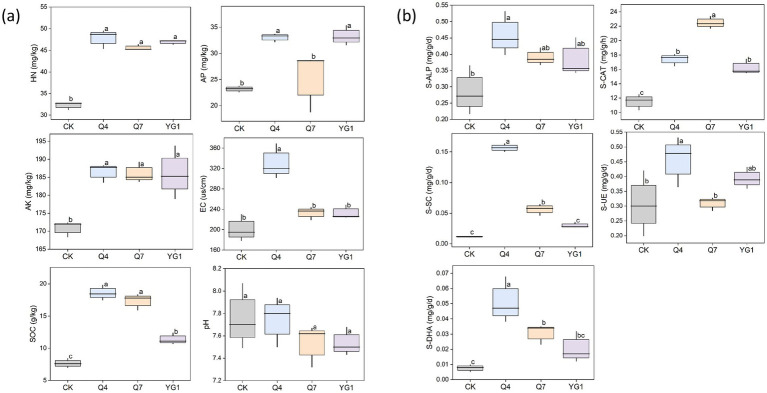
Effects of different treatments on the physical and chemical properties and enzyme activities of rhizosphere soil of plants. **(a)** soil nutrients. **(b)** soil enzymes. Letters indicate significant differences (*p* < 0.05) among different treatments.

The content of soil nutrients is closely related to plant growth and development, and plays a crucial role in determining soil fertility (Figure 7A). The contents of HN, AP, AK, electrical conductivity (EC), and SOC were significantly higher in the microbial agent treatment groups compared with the CK group (*p* < 0.05) (Supplementary Table S1). Specifically, the HN content increased by 48.19% and the AP content by 43.55%. The SOC content showed the most significant increase under the Q4 treatment (142.77%) and the smallest increase under the YG1 treatment (48.9%). Soil EC also exhibited varying degrees of increase, with Q4 treatment showing a 64.26% increase, Q7 a 15.92% increase, and YG1 a 15.87% increase.

Soil enzyme activity is a key indicator of soil fertility. The bacterial agent treatments significantly enhanced soil enzyme activities (*p* < 0.05) (Figure 7B). The S-CAT and S-ALP activities increased by 50.99 and 94.88%, and 61.38 and 37.44%, respectively, under the Q4 and Q7 treatments compared with the CK treatment. In addition, soil invertase (S-SC), urease (S-UE), and dehydrogenase (S-DHA) activities were significantly higher under the Q4 treatment compared with the CK treatment (*p* < 0.05), with increases of 1327.27, 49.67, and 537.50%, respectively. No significant changes were observed in the other treatment groups.

**Figure 8:**
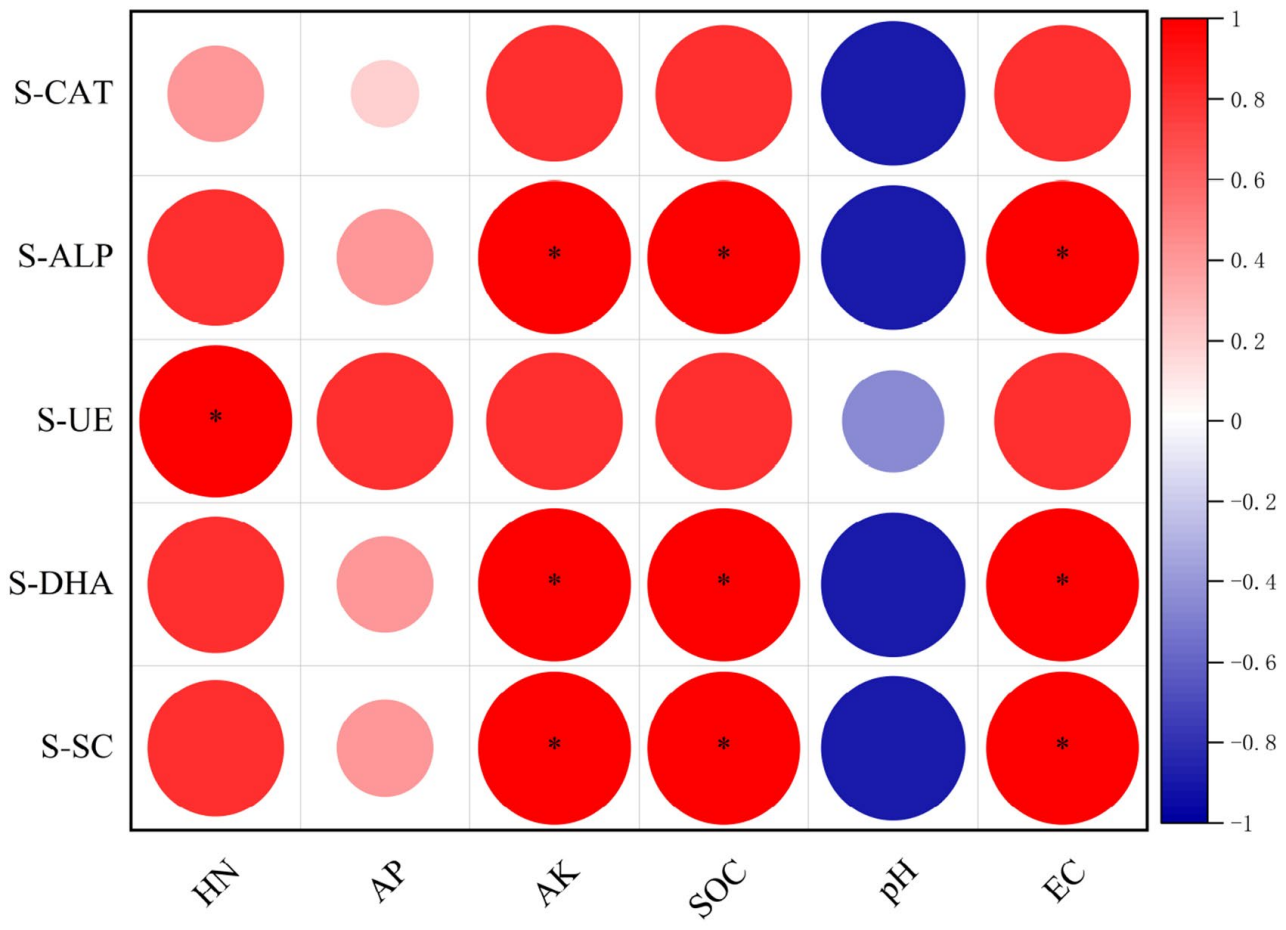
Correlations between soil enzyme activities and physicochemical properties. Significance levels: ^*^p < 0.05, ^**^p < 0.01, ^***^p < 0.001.

The correlation analysis between soil enzyme activities and physical and chemical properties revealed that S-CAT activity in *L. bicolor* Turcz soil (Figure 8) was positively correlated with HN, AP, AK, and SOC contents and EC, and negatively correlated with pH. S-ALP, S-DHA, and S-SC activities were significantly positively correlated with AK and SOC contents and EC (*p* < 0.05), positively correlated with the HN content, and negatively correlated with pH. Additionally, S-UE activity was significantly positively correlated with HN content (*p* < 0.05), positively correlated with AP, AK, and SOC contents and EC, and negatively correlated with pH.

### 3.4 Microbial community diversities and structure

This study specifically focused on strains with the stongest plant growth–promoting effects on *L. bicolor*. Results from the potted plant experiment indicated that the Q4 treatment exhibited the most pronounced growth-promoting effect on *L. bicolor*.

The bacterial community of rhizosphere soil samples was analyzed using 16S high-throughput Illumina MiSeq sequencing technology. A total of 532,543 bacterial sequences were obtained from all samples, which were clustered into 38,164 OTUs. The OTU dilution curve of the samples tended to be flat, indicating that the high-quality sequencing data were reasonable and reliable (Supplementary Figure S4). Table 3 shows that the Chao1, ACE, Shannon, and Simpson indices in the bacterial agent treatment group were lower than those in the CK group, indicating that the microbial agent application could reduce the richness of the potted community and make the species categories of soil microbial flora more concentrated to form a dominant group.

**Table 3:** Diversity indices of potted soil.

Treatment	Chao1	ACE	Sobs	Shannon	Simpson	Goods-Coverage
CK-1	1007.460	1046.655	941	7.876	0.988	0.990
CK-2	909.541	956.334	846	7.892	0.991	0.990
CK-3	884.328	918.849	830	7.923	0.990	0.990
Q4-1	887.961	929.449	823	7.551	0.986	0.990
Q4-2	882.573	919.364	831	7.729	0.989	0.991
Q4-3	936.938	975.488	860	7.534	0.986	0.990

**Figure 9:**
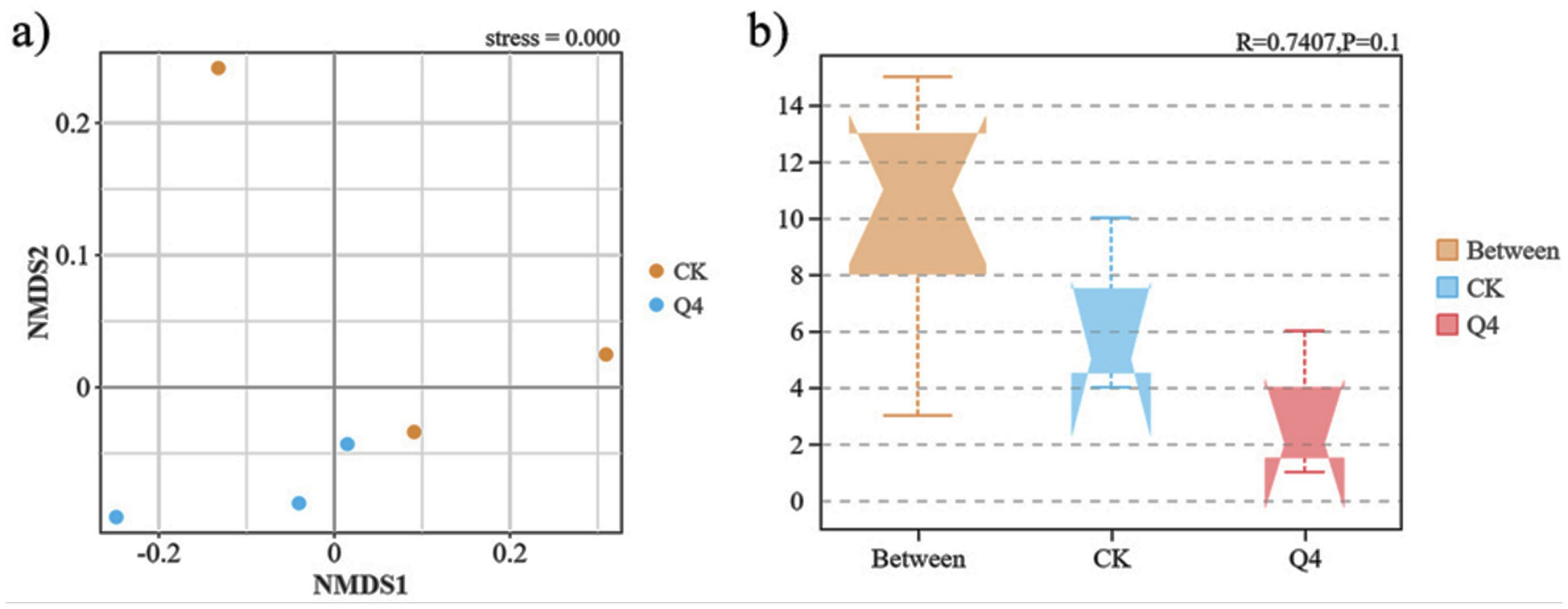
Beta diversity index of *Lespedeza bicolor* Turcz potting soil. **(a)** Non-metric multidimensional scaling (NMDS) analysis; **(b)** Analysis of similarities (ANOSIM) test.

Further, the beta diversity analysis (Figure 9A) revealed that the CK and Q4 treatments were separated along the non-metric multidimensional scaling (NMDS) axis. Part of the CK treatment was distributed on the positive axis of NMDS1. In contrast, the Q4 treatment was distributed on the negative axis of NMDS1, indicating that the soil bacterial community structure was significantly different after PSB treatment. The CK and Q4 treatments were separated along the NMDS2 axis. The CK treatment was distributed on the positive axis of NMDS2, and the Q4 treatment was distributed on the negative axis of NMDS2. This further confirmed the impact of PSB on the bacterial community structure of *L. bicolor* Turcz potted soil. The analysis of similarities (ANOSIM) test (Figure 9B) revealed that the between-group distance (Between) was greater than the within-group distance among soil samples under each treatment (*R* = 0.7407). Therefore, the composition of the bacterial community in *L. bicolor* Turcz rhizosphere soil varied among treatments.

**Figure 10:**
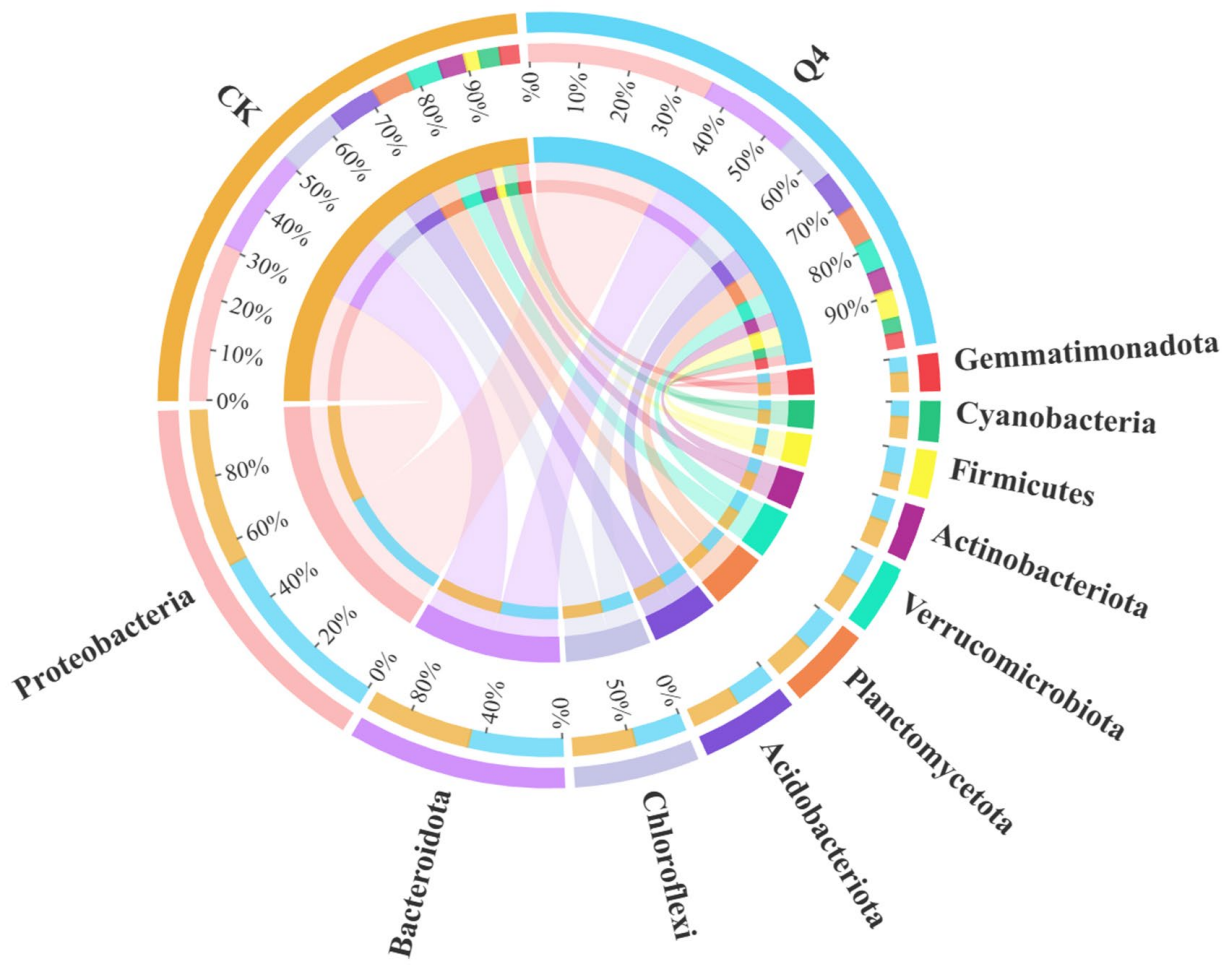
Stacked plot of phylum horizontal species distribution.

**Figure 11:**
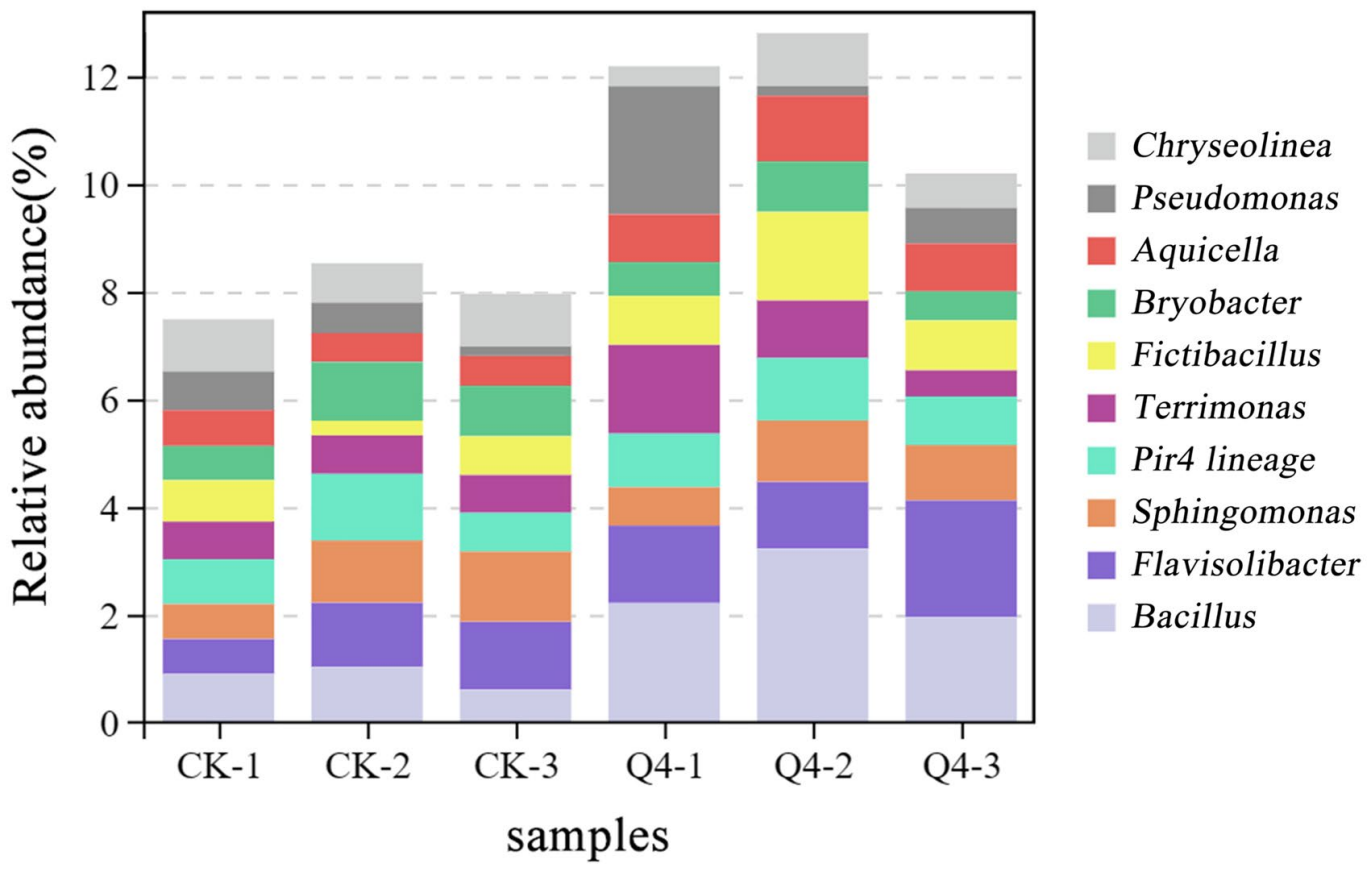
Stacked plot of genus horizontal species distribution.

The shifts in bacterial (phylum and genus) community composition across all treatments were investigated based on the hypervariable V3–V4 region (16S rDNA) during the 16S rRNA gene sequencing. Figures 10, 11 demonstrate the bacterial community at the phylum and genus levels in the soil samples. Of these phyla, Proteobacteria, Bacteroidota, Chloroflexi, Acidobacteriota, Planctomycetota, Verrucomicrobiota, Actinobacteriota, Firmicutes, Cyanobacteria, and Gemmatimonadota were the dominant phyla in bacterial communities (relative abundance of these species exceeding 1% in all treatments). The abundance of Proteobacteria and Firmicutes significantly increased, by 17.63 and 87.40%, respectively, under the Q4 treatment compared with the CK treatment.

The species changes were analyzed in the rhizosphere soil bacterial communities at the genus level. Compared with CK, the use of Q4 microbial agents increased the abundance of *Bacillus, Flavisolibacter, Terrimonas, Pseudobacillus,* and *Aquicella,* and the relative abundance increased from 0.84 to 2.47%, 1.03 to 1.63%, 0.59 to 1.16%, and 0.49 to 1.07% and 0.49 to 1.07%. In general, inoculation with the Q4 microbial agent significantly improved the soil colony structure and influenced the relative abundance of dominant microbial groups. Among these, the significant increase in the relative abundance of *Bacillus* and *Fictibacillus* may be an important factor in promoting plant growth.

### 3.5 Environmental factor analysis

**Figure 12:**
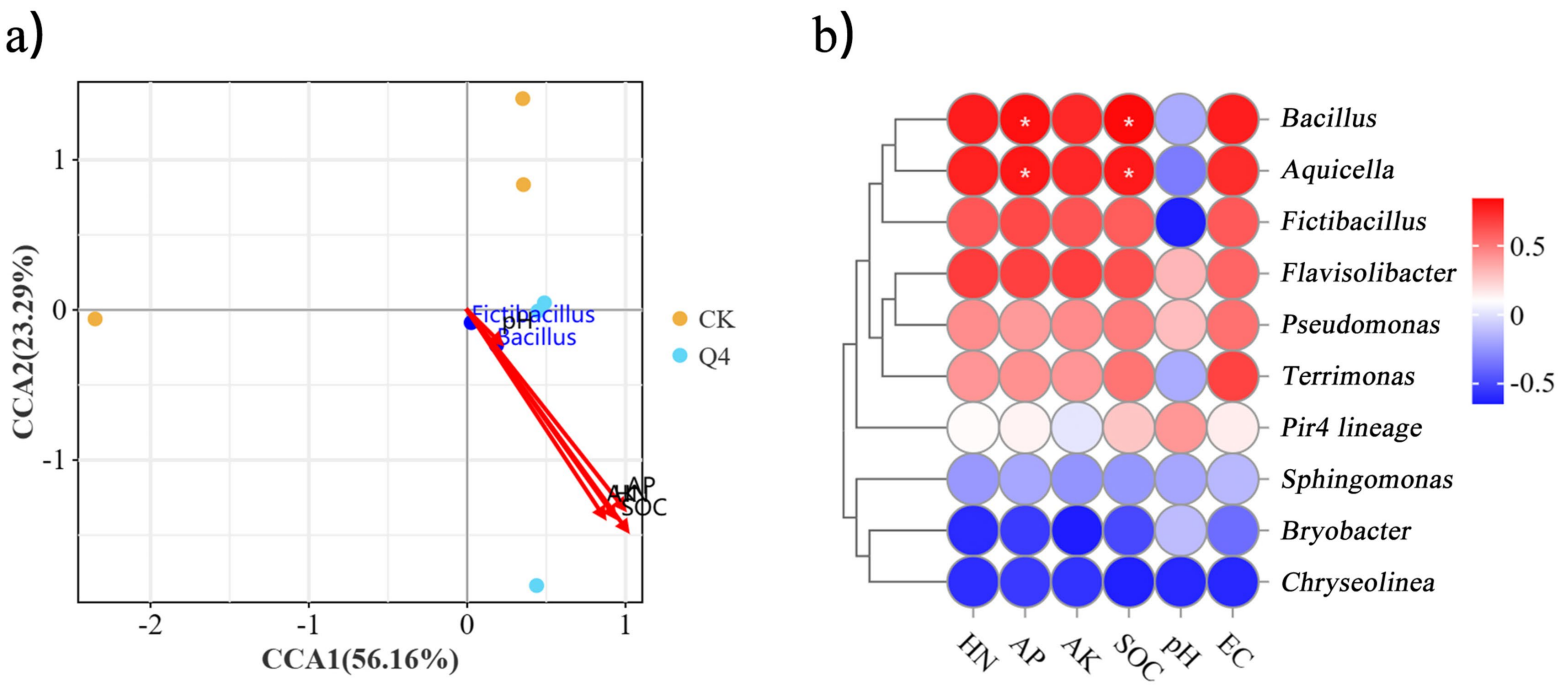
Analysis of *Lespedeza bicolor* Turcz potted bacterial community and environmental factors. **(a)** CCA analysis; **(b)** correlation heat map.

The results of the canonical correspondence analysis (CCA) showed that the samples in the CK and Q4 groups were aggregated separately. The abundance of *Bacillus* had an obvious correlation with environmental factors in the soil. CCA1 and CCA2 together explained 79.45% of the changes in bacterial community structure. The environmental factors SOC, EC, pH, AK, AP, and HN were positively correlated with the Q4 treatment (Figure 12A), indicating that the inoculation of microbial agents helped improve the release of soil nutrients. As shown in Figure 12B, the abundance of *Bacillus* was significantly positively correlated with AP and SOC contents (*p* < 0.05), positively correlated with HN, AK, and EC, and negatively correlated with pH (*r*^2^ = 0.03). The results suggested that *Bacillus* might be one of the potential reasons for Q4 to improve soil fertility and exert its growth-promoting effects.

### 3.6 Functional prediction

**Figure 13:**
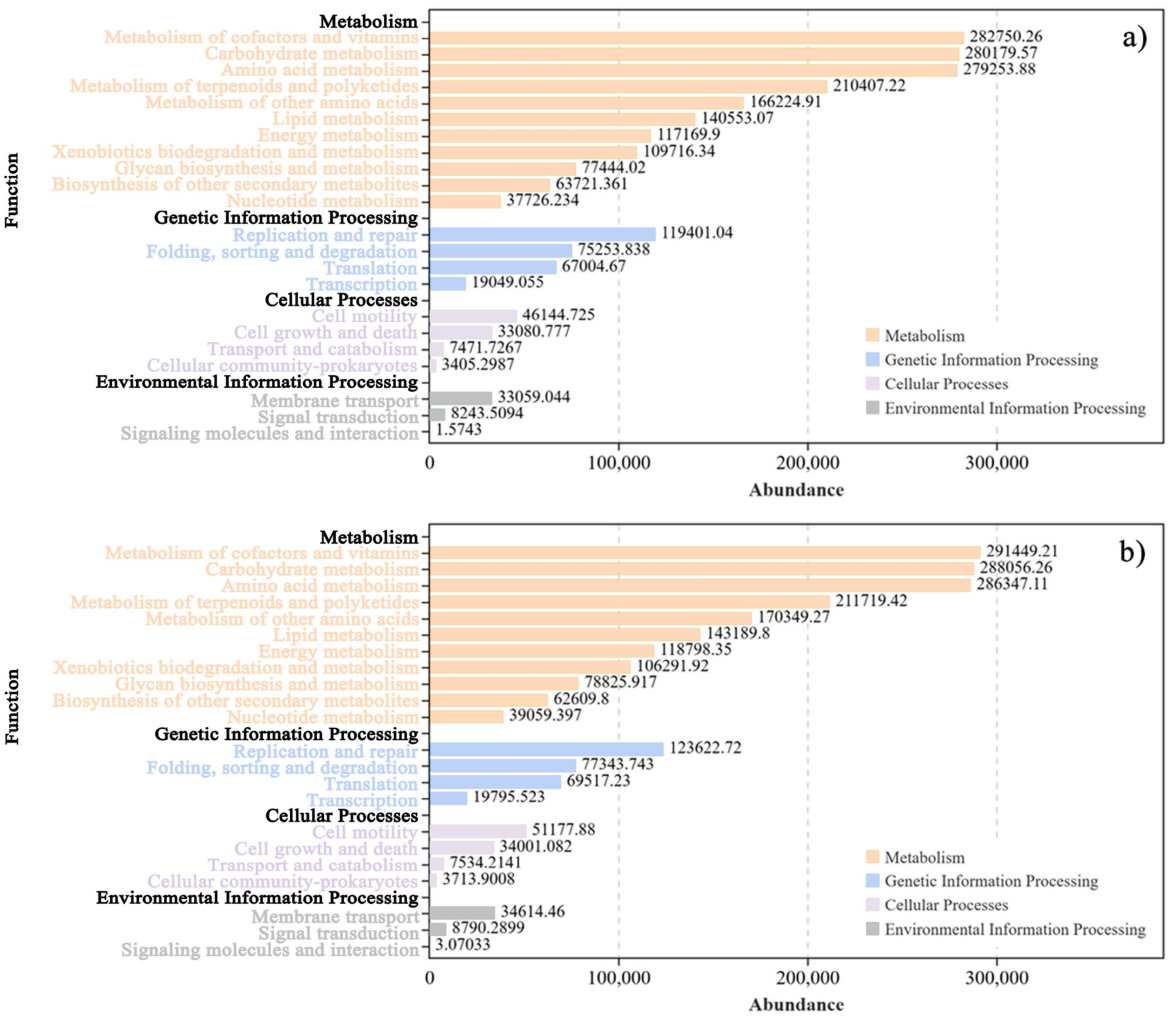
Prediction of functions under different treatments in *Lespedeza bicolor* Turcz based on KEGG database **(a)** CK; **(b)** Q4.

**Figure 14:**
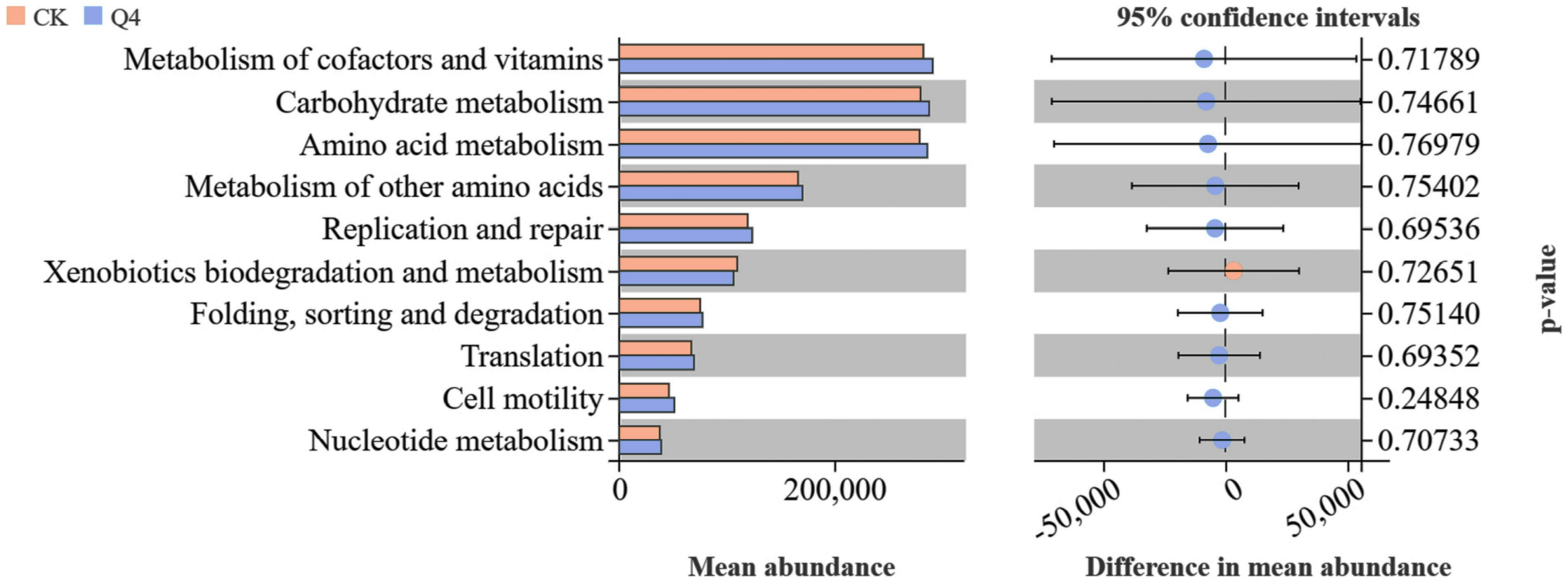
Welch’s *t* test results for *Lespedeza bicolor* Turcz.

The functional transformation of microbial communities is intrinsically linked to environmental factors. KEGG functional pathway analysis of *L. bicolor* Turcz soil microbial metabolites (Figure 13) revealed significant differences in the relative abundance of bacterial functional genes in *L. bicolor* Turcz soil samples under different treatments. The relative abundance of genes related to metabolism, genetic information processing, cellular processes, and environmental information processing in the Q4 treatment group was higher than that in the CK group, with metabolic genes showing the highest relative abundance. Welch’s *t* test (Figure 14) showed that the abundance of secondary functional genes related to cofactors and vitamins, carbohydrate metabolism, and amino acid metabolism increased under the Q4 treatment compared with the CK treatment.

## 4 Discussion

### 4.1 Phosphate-solubilizing capacity of bacterial strains

The present study demonstrated that all bacterial strains isolated from Qinghai–Tibet Plateau soils belonged to the genus *Bacillus*, which aligned with the findings of Zhiyuan et al. (2021) reporting *Bacillus* as a dominant genus in extreme soil environments of the plateau.

The phosphate-solubilizing capacity of these strains exhibited significant temperature dependence, with no growth observed at temperatures below 4 °C but robust growth and efficient phosphate solubilization at temperatures of 16–40 °C, indicating their thermal adaptability over a wide temperature range. This result may be because increasing the temperature increased the production of organic acids which decrease the pH of the solubilization medium, increasing P solubilization (Whitelaw, 2000; Barroso et al., 2006). Among these strains, Q4 (*B. atrophaeus*) exhibited optimal Po solubilization (190.36 mg L^−1^) at 30 °C, whereas Q7 (*B. megaterium*) showed peak Pi dissolution (132.39 mg L^−1^) at the same temperature, consistent with the findings of Mahdi et al. (2021), indicating that the optimal temperature for bacteria is 30 ± 2 °C. The three strains exhibited organic phosphorus solubilization capacities ranging from 110.56 to 170.80 mg mL^−1^ at 22 °C, with phosphorus solubilization efficiencies of 82.94–89.72% relative to their maximum capacities observed at 30 °C. Notably, Q4 showed significantly higher solubilization capacity compared to Q7 and YG1 (*p* < 0.05). These results demonstrate that strains can maintain high phosphorus-solubilizing capacity under low-temperature conditions, indicating strong cold adaptability. YG1 could grow at 16 °C–40 °C, but its ability to dissolve Po was lower at 30 °C than at 35 °C, indicating that it was more suitable for high-temperature environments. This might be because *B. toyonensis*, a new species of *B. cereus*, has a stronger ability to produce auxin and cytokinin and exhibits greater adaptability to extreme environments. In addition, Q4, Q7, and YG1 strains were highly active in phosphate solubilization, nitrogen fixation, and iron carrier production, respectively, and had good growth-promoting characteristics.

### 4.2 Internal mechanism of action of the strain on *L. bicolor* Turcz growth and soil enzyme activity

This study found that inoculating three PSB strains improved the seedling biomass, and root indices to varying degrees. This phenomenon may be attributed to the ability of these three PSB strains to solubilize and release insoluble P from soil, thereby enhancing phosphorus uptake efficiency and promoting plant growth (Rafique et al., 2017). Ashaduzzaman et al. (2016) demonstrated that adding PSB significantly enhanced the availability of nitrogen and P in paddy soil, promoted rice growth, and increased yield. Cruz and Ishii (2012) found that the inoculation of PSB promoted the root development of *Prunus mume*, enhanced soil P availability, and improved plant P absorption. In this study, the P content in *L. bicolor* Turcz seedlings inoculated with Q4 inoculant significantly increased by 33.07% compared with that in the group without microbial inoculant. Additionally, the growth-promoting effect was enhanced, indicating that the Q4 inoculant could promote the growth of plant seedlings by altering soil nutrient content. Specifically, Q4 inoculation increased the absorption of soil nutrients such as N, P, and K by plants, thereby increasing their concentrations in plant tissues. This improvement enhanced the regulation of stomatal conductance and transpiration, ultimately affecting the water status of seedling leaves (Mengel and Kirkby, 1980).

The results of this study showed that PSB inoculation increased chlorophyll content in the leaves of plant seedlings. This finding was consistent with the results of Deng et al. (2013), who reported that applying biofertilizers increased leaf chlorophyll content. Increased chlorophyll content improved the photosynthetic capacity of plants, leading to increased synthesis of organic matter, and, ultimately, enhanced tomato biomass. Sahu et al. (2023) found that the application of NPK + effective microorganisms (EM) + PSB significantly increased the chlorophyll content in soybean leaves, thereby increasing the soybean yield. The content of carotenoids in the leaves of *L. bicolor* Turcz seedlings significantly increased under different treatments. Carotenoids are essential endogenous antioxidants in plants (Borowiak et al., 2021). The increase in carotenoid content was conducive to the resistance of *L. bicolor* Turcz seedlings to various stresses. The photosynthetic parameters of plant leaves also increased to varying degrees with the application of PSB (Table 2). The application of biofertilizers can increase the stomatal conductance, transpiration rate, WUE, and intercellular CO_2_ concentration of leaves, thereby improving the net photosynthetic rate (Pn) of plants and promoting plant growth (Sheng et al., 2008). The experimental results of this study also supported this finding.

As bioactive substances with catalytic function, a large proportion of soil enzymes are secreted by microorganisms, animals, and plants, and their activity is an essential indicator of the content of soil nutrients (An et al., 2022). In this study, soil enzyme activity significantly improved following PSB inoculation because PSB inoculation significantly increased the organic matter content—an essential carbon source for soil microorganisms. This promoted microbial reproduction and activity. The substrate concentration of enzyme-catalyzed reactions also increased, implying that more enzymes could bind to substrates and enhance catalytic reaction rates, further improving soil enzyme activity.

Daniel et al. demonstrated that the application of nitrogen-fixing and PSB could significantly improve the activity of soil phosphatase and effectively promote the transformation of soil P (Torres-Cuesta et al., 2023). The results showed that the three strains of PSB could significantly improve the activity of S-ALP, with Q4 treatment having the most significant effect on *L. bicolor Turcz* seedlings. The ALP activity directly influences the decomposition and transformation of P in soil (Sardans et al., 2008). Therefore, the treatment with higher ALP activity in this study led to a higher AP content.

CAT activity also significantly increased in soils inoculated with the PSB inoculant, consistent with the findings of Yongqi et al. (2020), who showed that *B. subtilis* inoculation enhanced the activities of CAT and other enzymes in maize soil. CAT could remove harmful substances such as hydrogen peroxide from soil and played a critical role in plant growth and stress response. S-UE could catalyze the hydrolysis of organic nitrogen compounds to ammonia and played a crucial role in the soil nitrogen cycle. Its activity reflected the supply of soil nitrogen (Ni and Pacholski, 2022). In this study, S-UE displayed a promoting effect after inoculation with different PSB, which was positively correlated with soil nitrogen content. The S-SC activity was closely related to the mineralization rate of SOC, reflecting the degree of soil maturation and the level of soil fertility to a certain extent (Ji et al., 2014). In this study, the S-SC activity of PSB inoculant increased, which was consistent with the research conclusion of Sun et al. (2019). That is, the S-SC activity after treatment with *Trichoderma harzianum* and *Paenibacillus polymyxa* was the highest, which was conducive to improving soil fertility. Also, a series of biochemical reactions closely related to improvements in plant growth indicators and physical and chemical properties of the soil occurred. This study demonstrated a significant positive correlation between enzyme activity and soil nutrient content, consistent with previous research results (Torres-Cuesta et al., 2023). It indicated a key role of soil enzymes in soil nutrient transformation. The improvement in enzyme activity indicated an increase in soil nutrient transformation efficiency, an increase in soil available nutrient content, an improvement in soil fertility level, and promotion of plant growth.

In summary, Q4 (*B. atrophaeus*) outperformed Q7 and YG1 in promoting the growth of *L. bicolor* Turcz. This is probably because the host plants show selectivity when adsorbing external bacteria, or because the growth-promoting bacteria and host plants possess structural and functional specificity. This suggests the existence of an optimal host–microbe relationship.

### 4.3 Effects of inoculants on rhizosphere soil microorganisms of *L. bicolor* Turcz

Inoculation with beneficial microorganisms is one of the essential factors influencing the rhizosphere microbial community (Micallef et al., 2014). For example, PSB can promote the growth and development of plants by interacting with plant roots. They also directly or indirectly impact the structure of the soil microbial community and enhance the stability of the soil microbial ecosystem, ultimately influencing the functional potential of associated microbiota (Thokchom et al., 2017). In this study, the Chao1, ACE, Shannon, and Simpson indices of *L. bicolor* Turcz rhizosphere soil bacterial community decreased after PSB inoculation, which was consistent with the findings of Zhang et al. (2024). That is, the combined application of biofertilizers reduced the microbial community abundance in grassland rhizosphere soil. Beta diversity analysis showed that the bacterial community structure in the rhizosphere of *L. bicolor Turcz* was significantly altered after PSB inoculation, indicating that the addition of exogenous microbial agents markedly impacted the community structure of soil microorganisms. This finding was consistent with the research results of Anestis et al., who reported that the addition of rhizosphere growth-promoting bacteria A1501 significantly changed the local soil bacterial community structure (Karkanis et al., 2018).

The composition of the soil microbial community reflects not only its biogeochemical cycling capacity but also its impact on soil fertility (Manasa et al., 2020; Moreira et al., 2020). This study found that Proteobacteria and Bacteroidota accounted for the highest proportion, which was consistent with the findings of Constantine et al. (2022) on the composition of the bacterial community in the rhizosphere soil of *Lycium barbarum*. That is, these bacteria may be common in the rhizosphere soil microbial community. Studies have shown that most members of these phyla usually display growth-promoting characteristics, and the inoculation of PSB can improve the relative abundance of beneficial microorganisms in soil (Zheng et al., 2020). Proteobacteria and Bacteroidota are typically copiotrophic phyla, whose relative abundance increases with soil nutrient availability. Bacteroidota has the effect of dissolving P, which is positively correlated with the AP content (Lidbury et al., 2020). The results of this study were consistent with previous findings. The relative abundance of Firmicutes in the PSB-inoculated group was significantly higher than that in the CK group, which was due to the inoculated PSB strain being a *Bacillus* species, belonging to the phylum Firmicutes; moreover, the relative abundance of Firmicutes increased with the increase in soil nutrient content (Liu et al., 2019).

The physicochemical properties of soil are the key determinants of microbial community structure (Chen et al., 2016; Marschner et al., 2001). Wang et al. (2023) demonstrated significant effects of SOC and AK contents on soil microorganisms. Wang et al. (2018) found pH, SOM, and AP as important factors influencing the bacterial community in maize rhizosphere. In this study, the CCA analysis of microbial community structure also reached a similar conclusion. That is, SOC, AK, AP, HN, and EC had a significant impact (*p* < 0.05, Figure 11) on soil microbial community structure. The contents of AP, AK, HN, and SOC in *L. bicolor* Turcz soil increased significantly with the addition of the Q4 strain. This influenced the abundance of beneficial soil microorganisms, promoting the release of nutrients in the soil that were conducive to plant absorption and utilization, and thus promoting plant growth. The correlation analysis also revealed that the difference in soil bacterial community structure was significantly positively correlated with the difference in soil physical and chemical properties. This indicated that the physical and chemical properties and bacterial structure of soil were interactive and interdependent, indicating the interaction between soil microorganisms and plants. In contrast, the response of *L. bicolor* Turcz soil microbial community to the changes in environmental factors was not significant. This might be because *L. bicolor* Turcz had stronger environmental adaptability, or its soil microbial community itself had high stability, effectively resisting the fluctuations in environmental factors.

The microorganisms involved in soil nutrient transformation are interconnected through gene-level interactions (Chen et al., 2019; Srour et al., 2020). Some studies have highlighted that the metabolic function is the main function of the soil bacterial community participating in the biogeochemical cycle (Pan et al., 2020). For example, carbohydrate metabolism is closely related to nitrogen fixation and P release, which can promote the absorption of nitrogen and P by plant roots (Liu et al., 2021). Additionally, some metabolic processes can also produce antibiotics and hormones, promoting plant growth and development (Halifu et al., 2019). In this study, the PICRUSt2 method was used to infer differences in functional traits of the microbial community through *16S rRNA* gene data. The relative abundance of genes related to metabolism, genetic information processing, environmental information processing, and cellular processes under the primary biological metabolic pathway increased in the microbial inoculant treatment group, with a notable increase observed in metabolism-related genes. The metabolic processes mainly included carbohydrate metabolism, amino acid metabolism, cofactors, and vitamins. The relative abundance of genes in these metabolic pathways significantly increased, especially under treatment with microbial agents. These metabolic processes can provide a carbon source to further promote the symbiotic nitrogen fixation in legumes or facilitate the degradation of amino acids to auxin and other growth-promoting substances for plant roots and shoots. In addition, cofactors can promote enzymatic reactions, and vitamins have a positive efefct on the growth and development of plants. Microbial agents can significantly increase the gene abundance of these metabolic pathways, indicating their vital role in plant responses to environmental changes, especially in interactions with exogenous microbial agents. They may serve as an important factor in regulating plant growth.

## 5 Conclusion

This study demonstrated that inoculation with *Bacillus atrophaeus* (Q4) significantly enhanced the growth of *Lespedeza bicolor* Turcz. by modulating soil microbiota and improving soil fertility. The Q4 strain elevated key soil nutrient contents and increased the activity of urease, alkaline phosphatase, catalase, and sucrase, thereby improving overall soil quality. These changes were associated with a marked increase in plant biomass and root system development compared to the non-inoculated controls (CK). Mechanistically, the Q4 inoculant stimulated plant growth by enriching beneficial bacterial communities (e.g., *Bacillus*, *Flavisolibacter*, and *Pseudomonas*) and improved critical environmental factors including nitrogen, phosphorus, potassium availability and organic matter content. These findings provide valuable insights into how Q4 inoculation enhances plant adaptability and restoration capacity, suggesting that microbial-plant collaborative ecological remediation represents a promising strategy. Future studies should evaluate the practical application of ‘Q4 + *Lespedeza*’ model in the ecological restoration of high and steep slopes and other difficult places in high and cold regions, in order to develop efficient bioremediation technology.

## Data Availability

The datasets presented in this study can be found in online repositories. The names of the repository/repositories and accession number(s) can be found in the article/Supplementary material.
