# Supplementary material for: Isolation and growth-promoting mechanisms of phosphate-solubilizing bacteria from Qinghai-Tibet Plateau in Lespedeza bicolor Turcz
Source: Front Microbiol. 2025 Sep 17;16:1669774. doi: 10.3389/fmicb.2025.1669774 (PMC12484056; doi:10.3389/fmicb.2025.1669774)
Supplement: Supplementary file 1 [file Table_1.DOCX]

**Supplementary Materials**

## Media

*Luria broth* (LB, suitable for bacterial colonization): 10 g of peptone, 5 g of yeast extract, 5 g of NaCl, and 1,000 mL of deionized water, adjusted to pH 7.2.

*Monkina organic phosphorus* (for screening strains with the phosphorus-solubilizing effect):10 g of glucose, 0.5 g of (NH_4_)_2_SO_4_, 0.3 g of NaCl, 0.3 g of KCl, 0.3 g of MgSO_4_·7H_2_O, 0.03 g of FeSO_4_·7H_2_O, 0.03 g of MnSO_4_, 5.0 g of CaCO_3_, 0.3 g of lecithin, 20 g of agar, and 1,000 mL of deionized water, pH 7.0-7.5.

*Inorganic phosphorus* (for screening strains with the phosphorus-solubilizing effect): 10 g of glucose, 0.5 g of (NH_4_)_2_SO_4_, 0.3 g of NaCl, 0.3 g of KCl, 0.3 g of MgSO_4_·7H_2_O, 0.03 g of FeSO_4_·7H_2_O, 0.03 g of MnSO_4_, 5.0 g of Ca_3_(PO_4_)_2_, 20 g of agar, and 1,000 mL of deionized water, pH 7.0-7.5.

*Ashby’s nitrogen-free medium* (Nfb, for the culture of authigenic nitrogen-fixing bacteria and potassium bacteria): 10 g of mannitol, 0.2 g of KH₂PO₄, 0.2 g of MgSO_4_·7H_2_O, 0.2 g of NaCl, 0.2 g of CaSO₄·2H₂O, 5 g of calcium carbonate, 20 g of agar, and 1,000 mL of deionized water, pH 7.0-7.2.

1. *CAS* *medium* (CAS, for the detection of siderophore production by microorganisms): 60.5 mg of Chrome Azurol S (CAS), 72.9 mg of hexadecyltrimethylammonium bromide (HDTMA), 2,645 mg of FeCl₃·6H₂O, 295.25 mg of NaH₂PO₄·2H₂O, 1,213.5 mg of Na₂HPO₄·12H₂O, 125 mg of NH_4_Cl, 37.5 mg of KH₂PO₄, 62.5 mg of NaCl, 9,000 mg of agar, and 1,000 mL of deionized water, pH 6.8±0.1.

| 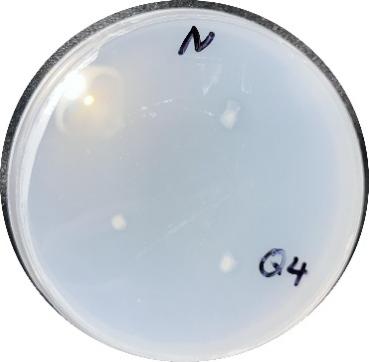 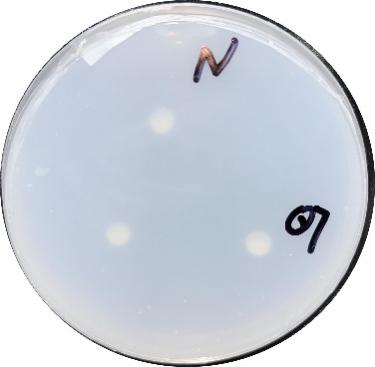 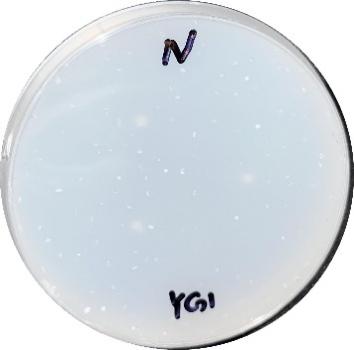  **Fig. S1** Effects of *Bacillus* *atrophaeus* Q4 (a)、*Bacillus megaterium* Q7 (b) and *Bacillus toyonensis*YG1 (c).  on nitrogen fixation.  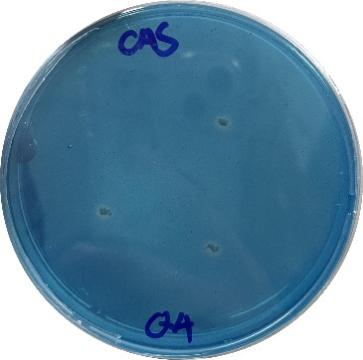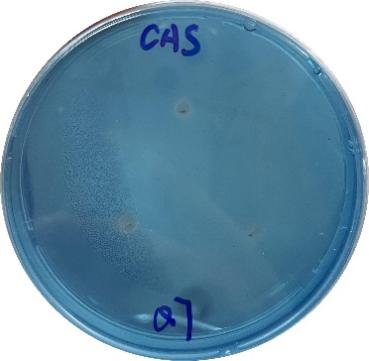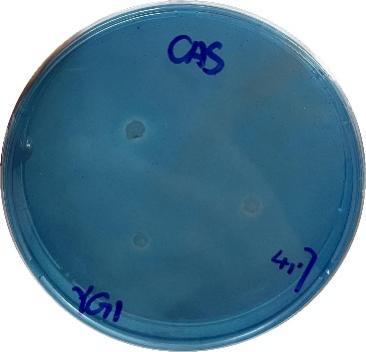  **Fig. S2** Effects of *Bacillus atrophaeus* Q4 (a)、*Bacillus megaterium* Q7 (b) and *Bacillus toyonensis*YG1 (c).  on siderophore secretion. |
| --- |
| \| 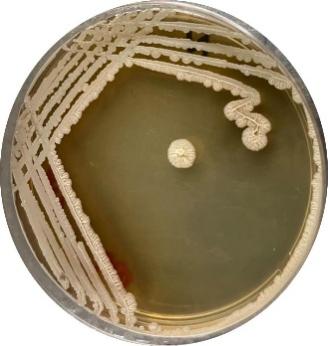 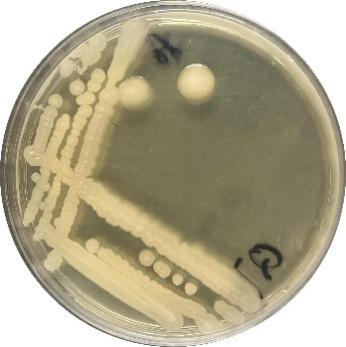 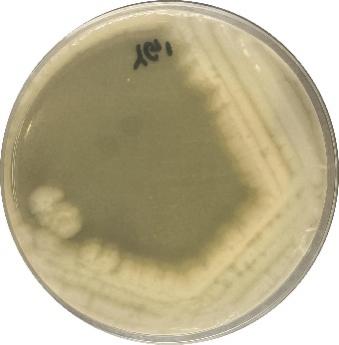 \| \| --- \|   **Fig. S3** Schematic diagram of *Bacillus atrophaeus* Q4 (a)、*Bacillus megaterium* Q7 (b) and *Bacillus toyonensis*YG1 (c). |

**Table S1.** Effects of different treatments on the physical and chemical properties of rhizosphere soil of plants

| **Treatment** | **HN（mg/kg）** | **AP（mg/kg）** | **AK（mg/kg）** | **EC (µs/cm)** | **SOE(g/kg)** | **pH** |
| --- | --- | --- | --- | --- | --- | --- |
| CK | 32.29 ± 0.90b | 23.18 ± 0.61b | 170.90 ± 2.22b | 201.07 ± 26.62b | 7.65 ± 0.75c | 7.75 ± 0.29a |
| Q4 | 47.85 ± 2.23a | 33.07 ± 0.84a | 186.53 ± 2.63a | 330.27 ± 34.88a | 18.57 ± 1.19a | 7.54 ± 0.13a |
| Q7 | 45.60 ± 0.66a | 25.32 ± 5.70b | 186.03 ± 2.93a | 233.07 ± 12.83b | 17.34 ± 1.30a | 7.54 ± 0.19a |
| YG1 | 46.88 ± 0.53a | 33.28 ± 1.93a | 186.00 ± 7.43a | 232.97 ± 9.06b | 11.39 ± 0.93b | 7.75 ± 0.22a |

| 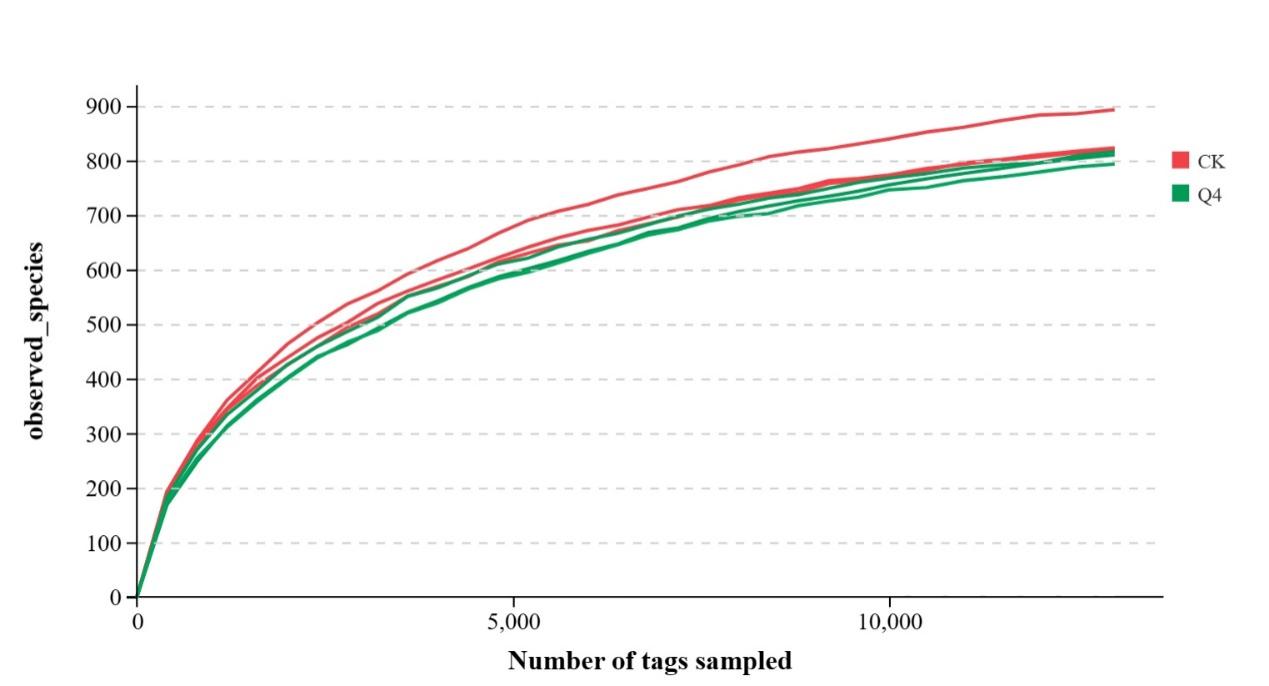 |
| --- |
| **Fig. S4** Variation in the dilution curves of rhizosphere soil bacteria under inoculantion with *Bacillus* *atrophaeus* （Q4） |


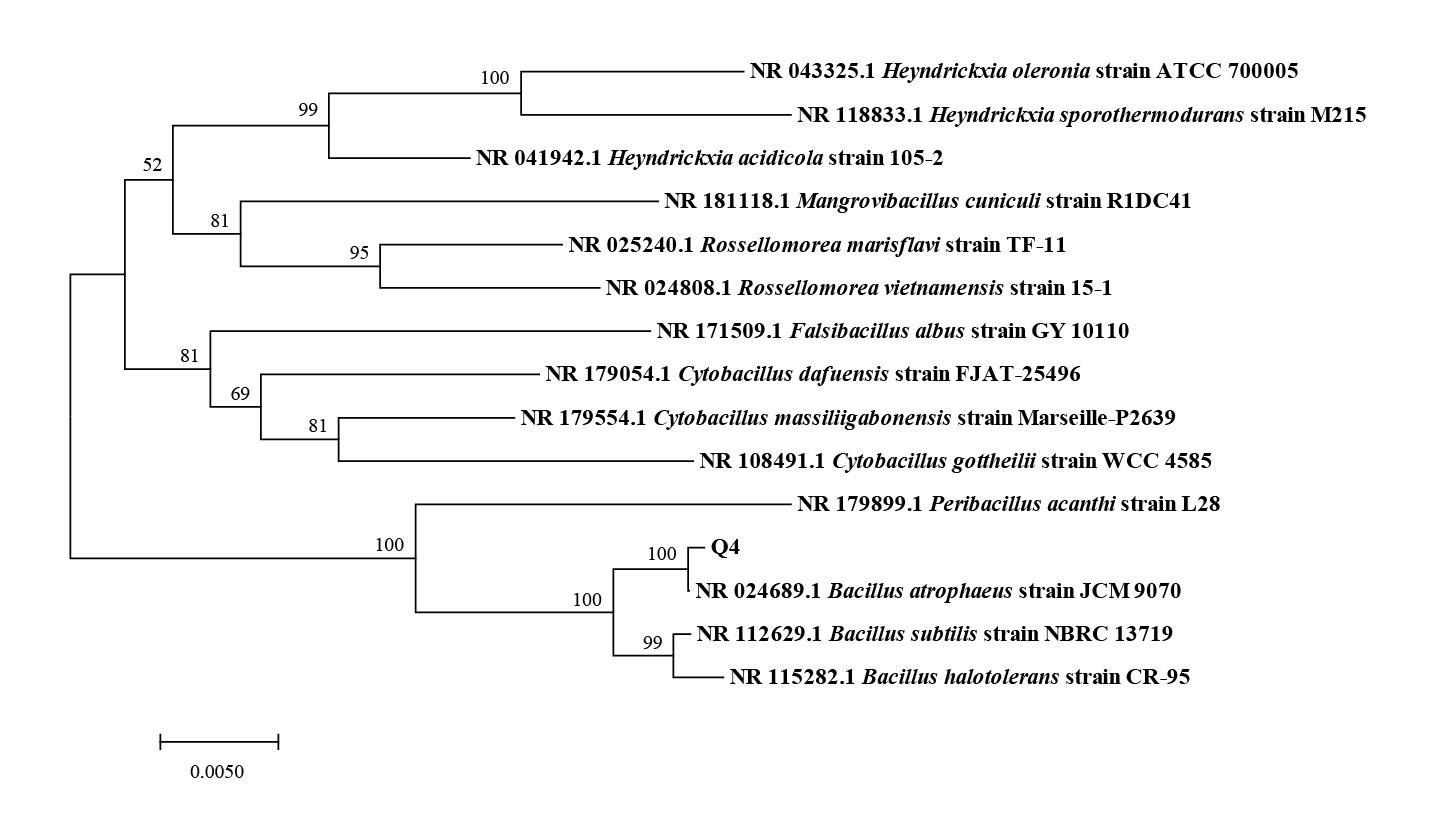


**Fig. S5** Phylogenetic tree of Q4 strains


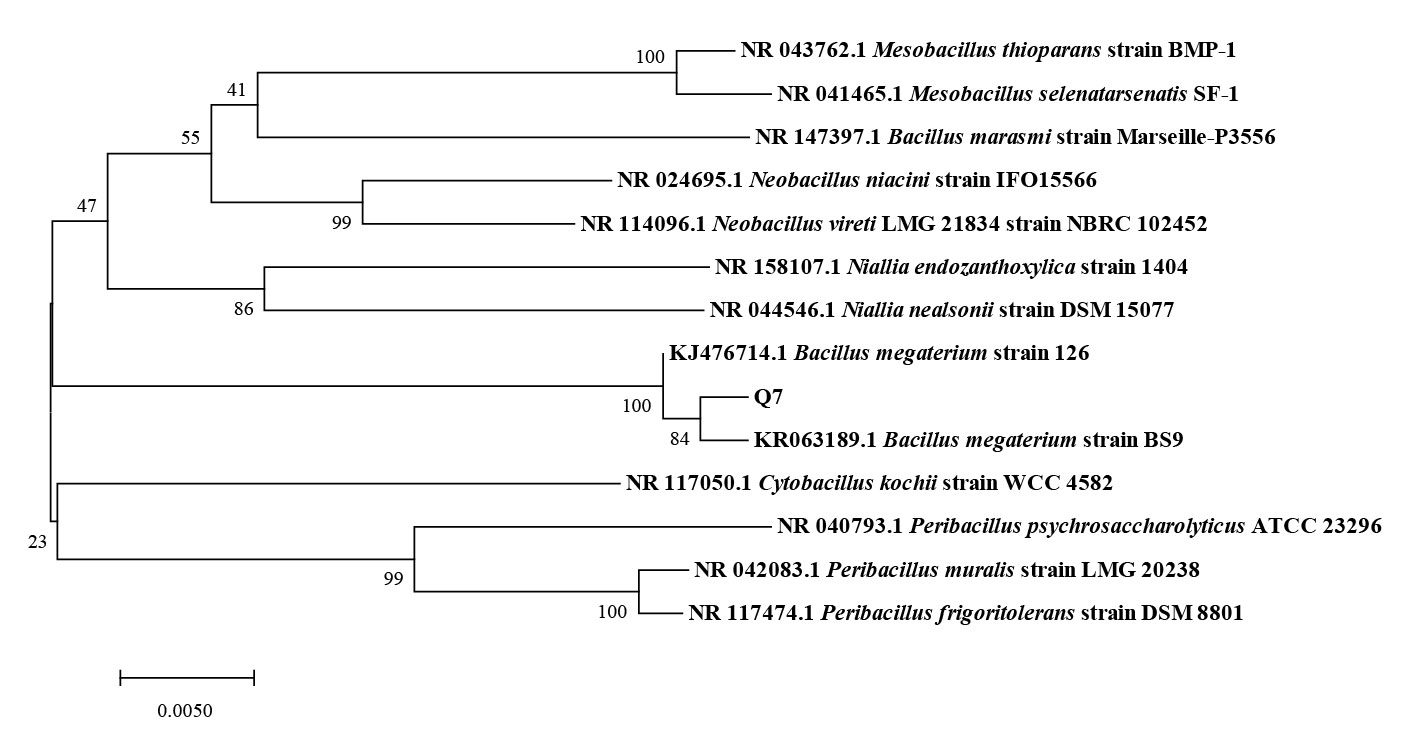


**Fig. S6** Phylogenetic tree of Q7 strains


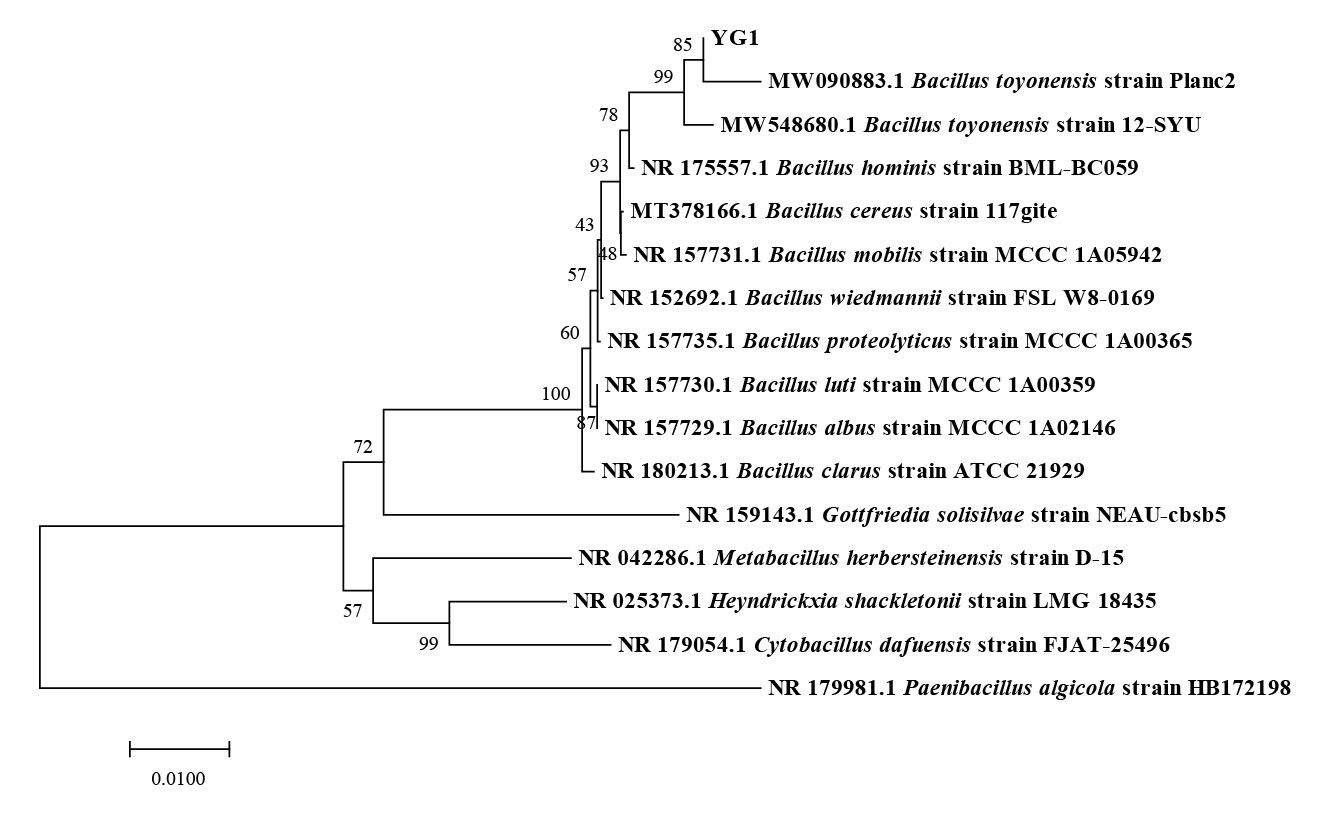


**Fig. S7** Phylogenetic tree of YG1 strains
